# Neuroprotective Action of Multitarget 7-Aminophenanthridin-6(*5H*)-one Derivatives against Metal-Induced Cell Death and Oxidative Stress in SN56 Cells

**DOI:** 10.1021/acschemneuro.1c00333

**Published:** 2021-08-30

**Authors:** Paula Moyano, David Vicente-Zurdo, Cristina Blázquez-Barbadillo, J. Carlos Menéndez, Juan F. González, Noelia Rosales-Conrado, Javier del Pino

**Affiliations:** † Departamento de Farmacología y Toxicología, Facultad de Veterinaria, Universidad Complutense, 28040 Madrid, Spain; ‡ Departamento de Química Analítica, Facultad de Ciencias Químicas, Universidad Complutense, 28040 Madrid, Spain; § Unidad de Química Orgánica y Farmacéutica, Departamento de Química en Ciencias Farmacéuticas, Facultad de Farmacia, Universidad Complutense, 28040 Madrid, Spain

**Keywords:** Metal cytotoxicity, neuroprotection, chelating activity, antioxidants, phenanthridones

## Abstract

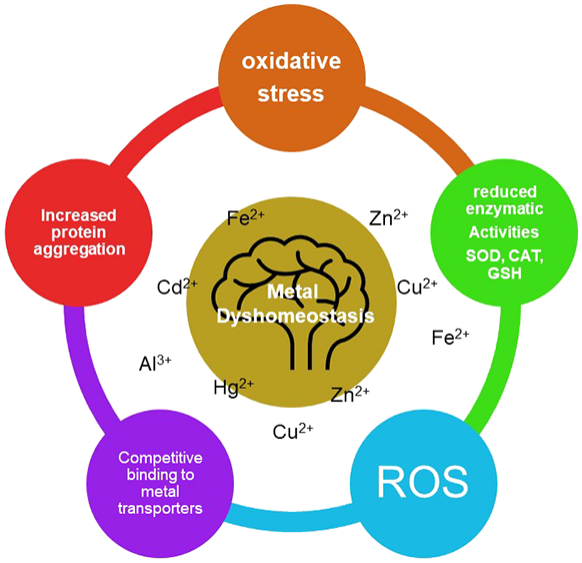

Neurodegenerative diseases have been associated with brain metal accumulation, which produces oxidative stress (OS), matrix metalloproteinases (MMPs) induction, and neuronal cell death. Several metals have been reported to downregulate both the nuclear factor erythroid 2-related factor 2 (Nrf2) pathway and the antioxidant enzymes regulated by it, mediating OS induction and neurodegeneration. Among a recently discovered family of multitarget 7-amino-phenanthridin-6-one derivatives (**APH**) the most promising compounds were tested against metal-induced cell death and OS in SN56 cells. These compounds, designed to have chelating activity, are known to inhibit some MMPs and to present antioxidant and neuroprotective effects against hydrogen peroxide treatment to SN56 neuronal cells. However, the mechanisms that mediate this protective effect are not fully understood. The obtained results show that compounds **APH1**, **APH2**, **APH3**, **APH4**, and **APH5** were only able to chelate iron and copper ions among all metals studied and that **APH3**, **APH4**, and **APH5** were also able to chelate mercury ion. However, none of them was able to chelate zinc, cadmium, and aluminum, thus exhibiting selective chelating activity that can be partly responsible for their neuroprotective action. Otherwise, our results indicate that their antioxidant effect is mediated through induction of the Nrf2 pathway that leads to overexpression of antioxidant enzymes. Finally, these compounds exhibited neuroprotective effects, reversing partially or completely the cytotoxic effects induced by the metals studied depending on the compound used. **APH4** was the most effective and safe compound.

## Introduction

Several neurodegenerative disorders including Alzheimer’s disease (AD), Parkinson’s disease (PD), Huntington’s disease (HD), and amyotrophic lateral sclerosis (ALS), among others, are undergoing a worrying increase in incidence worldwide. Although the ultimate causes of these diseases remain unknown, the possible involvement of environmental factors in their development has been suggested.^1−3^ The rise in human industrial activity has exponentially increased the population’s exposure to heavy metals and their body accumulation, leading to toxic effects.^1−4^ Brain accumulation of heavy metals, such as aluminum (Al), cadmium (Cd), and mercury (Hg) due to overexposure, or essential metals, including iron (Fe), copper (Cu) and zinc (Zn) due to pathological circumstances, has been correlated with the development of neurodegenerative diseases.^1−8^ Accumulation of metals in the brain was related with the induction of oxidative stress generation.^1,2^ Oxidative stress, which results from the mismatch between reactive oxygen species (ROS), reactive nitrogen species (RNS), and antioxidant defense can cause oxidative damage to cell structures and cell death.^1,2^ The central nervous system (CNS) is the most metabolically active organ, resulting in a high production of superoxide radical anion (O_2_^–^), hydroxyl radical (OH•), and hydrogen peroxide (H_2_O_2_), thus being particularly susceptible to oxidative stress and ROS damage.^1,2^ Furthermore, ROS generation was reported to mediate the brain accumulation of oxidized, misfolded, and aggregated proteins such as tau-phosphorylated (p-tau), α-synuclein (α-syn), and Aβ peptides. After their accumulation, they induce neurofibrillary tangles, amyloid plaques, and Lewy bodies, the main hallmarks of AD and PD, neuroinflammation, mitochondrial dysfunction, and finally neuronal cell death.^1,2^

Calcium and zinc regulate the activity of matrix metalloproteinases (MMPs), which control a variety of physiological processes. Under pathological conditions, MMPs overexpression or abnormal expression has been related with the induction of brain damage and with neurodegenerative disease, including AD, PD, HD, and ALS.^9^ Otherwise, heavy metals were also related to the induction of MMPs, which could lead to cytotoxicity.^10^ The main MMPs present in the brain are MMP-2/3/9/14, which play a key role in the neuroinflammatory processes and brain damage.^11,12^ Besides, MMPs dysfunction was related with the production of Aβ, p-tau, α-synuclein, and other abnormal proteins related with neurodegenerative diseases, especially MMP-9.^9,13−15^ Therefore, MMP inhibitors were tested to improve symptoms of different neurodegenerative diseases and to reduce the toxicity associated with metals.^16^

In this context, the control of metal accumulation in the CNS and its pathological effects is an important target in the development of new therapeutic agents against neurodegeneration and neurodegenerative disorders. In this sense, we have previously reported the discovery of new multitarget phenanthridin-6(*5H*)-one derivatives (APH) that are able to inhibit MMP enzymes, reduce oxidative stress, and protect against ROS.^17^ Although these compounds were designed to present chelating activity, computational studies indicated binding to a distal region at the S1′ site, with no involvement of the catalytic zinc ion.^16^ Thus, it is necessary to confirm experimentally the role of the Zn^2+^ ion in MMPs inhibition by these compounds.

Furthermore, the neuroprotective effects shown for these compounds against oxidative stress and cell death induced by metals could be mediated through a direct ROS scavenger activity. The induction of cellular antioxidant enzymes, which reduce ROS, may also be involved, since they show neuroprotective and antioxidant action against hydrogen peroxide in SN56 cells.^17^ Nuclear factor erythroid 2-related factor 2 (Nrf2) is a key factor in the transcription regulation of antioxidant cytoprotective enzymes such as superoxide dismutase 1, catalase, glutathione peroxidase, NAD(P)H quinone oxidoreductase 1, and heme oxygenase-1, among others.^18−20^ Dysfunction of the Nrf2 pathway has been identified, in different neurodegenerative diseases, as a factor of oxidative stress generation.^21−23^ Besides, many metals were reported to alter the regulation of the Nrf2 pathway, leading to oxidative stress and cell death induction.^24−27^ Nrf2 levels are regulated by kelch-like ECH-associated protein 1 (Keap1), which under normal conditions binds to Nrf2 in the cytoplasm, promoting its degradation. However, under oxidative stress, Keap1 is inactivated and releases Nrf2, which is transported to the nucleus, inducing the expression of downstream pathway antioxidant enzymes.^19,20^ Another mechanism to avoid the oxidative stress, and other harmful actions of metals, is to sequester them with chelator compounds. Thus, as these compounds were designed to present chelating activity, they could also protect against metals toxicity through this mechanism.

In the context of the treatment of neurodegenerative diseases, it is crucial to find new multitarget drugs that reduce the neurotoxic effects of metals that may avoid the onset and progression of these diseases. In this sense, the use of molecules designed to possess antioxidant action and as metal ionophores to sequester, redistribute, and remove metals in the CNS is an attractive therapy to reduce the neurotoxic effect of metals in the brain. To reach this aim, we studied the chelating activity, the action on Nrf2 pathway, and the neuroprotective action against heavy and essential metals of a small library of 7-amino-phenanthridin-6-one derivatives that had been previously characterized as MMP inhibitors and found to exert the most neuroprotection against oxidative stress.

## Results and Discussion

### Chemistry

**Scheme 1:**
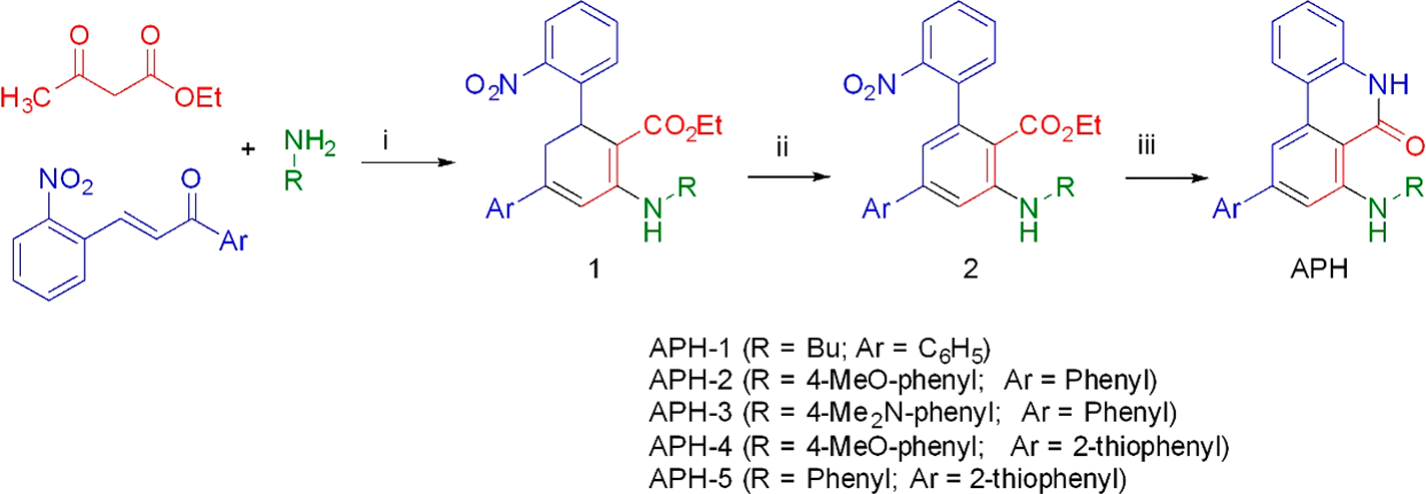
Synthesis of the Target Compounds **APH** Reagents and conditions: (i) EtOH, CAN (5%) reflux, 16 h; (ii) DDQ (1.5 equiv), toluene, 2 h at room temperature; (iii) EtOH, Pd/C (10%), H_2_ 1 atm, 2 h at room temperature.

The target compounds **APH1**–**APH5** were synthesized as shown in Scheme 1 by our previously reported method.^17^ Thus, 4,6-diaryl-5,6-dihydroanthranylate derivatives **1** were obtained by a CAN-catalyzed multicomponent reaction between *ortho*-nitrochalcone, ethyl acetoacetate, and the suitable primary amines. This was followed by oxidative dehydrogenation of the central ring in the presence of 2,3-dichloro-5,6-dicyano-1,4-benzoquinone (DDQ) and reductive cyclization to give the target compounds **APH1**–**APH5** in good overall yield.

### Metal-Chelating Properties of Compounds **APH1**–**APH5**

**Figure 1:**
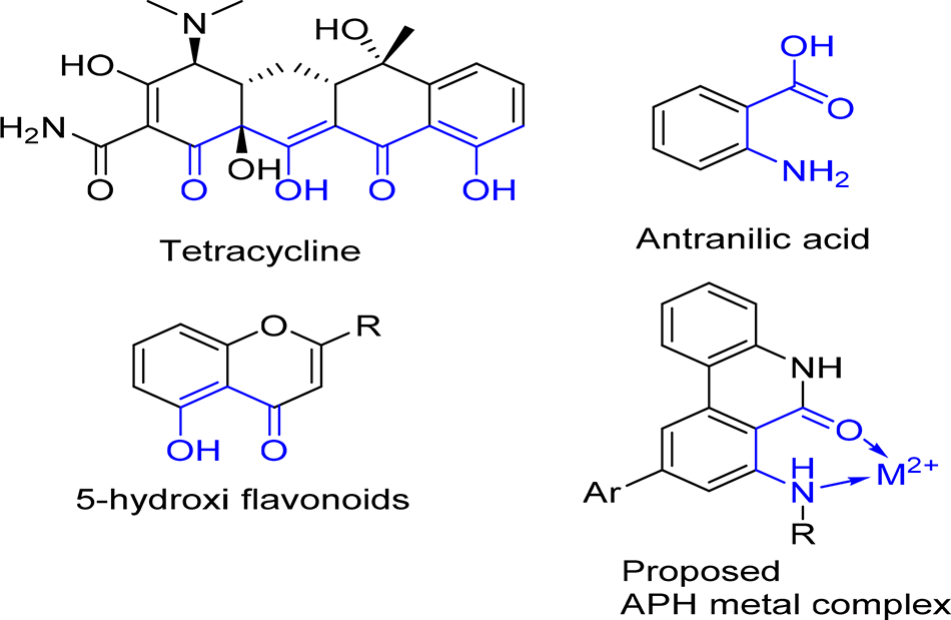
Hypothesized **APH**–metal interaction and some therapeutically useful chelating biomolecules.

N and O electron-donor atoms and carbonyl group assembled to a cyclic system are considered suitable for efficient interactions with metal cations. These binding moieties are present in some biological relevant compounds such as the tetracycline,^28^ 3-hydroxy flavonoids,^29^ and anthranilic acid,^30^ all of which have chelating properties. Thus, we designed the **APH** compounds as bidentate ligands to interact with the metal anions guest via a potential metal-binding site formed by the lactam group and the exocyclic nitrogen atom, hypothesizing that the substituents R on the nitrogen atom at C-7 position could modulate the affinity of the nitrogen atom for metals (Figure 1). For the current study, we selected compounds **APH1**–**APH5** from our library, due to their higher activity on the selected targets (i.e., MMPs inhibition and antioxidant activity), according to our previous studies.^17^

The ability of the **APH1**–**APH5** to bind Fe^2+^, Cu^2+^, Zn^2+^, Hg^2+^, Cd^2+^, and Al^3+^ was studied by ultraviolet–visible (UV–vis) spectroscopy. This is one of the most widely used techniques to study chelation and to elucidate the stoichiometry of the formed metal complexes.^31−34^ To this end, the UV–vis spectra of the compounds alone and in the presence of one of the evaluated metals were compared. Blank solutions composed of dimethyl sulfoxide (DMSO) 100 mM phosphate buffer at pH 7.30 showed high absorption values between 190 and 240 nm, and consequently only absorbances from 240 to 480 nm were considered.

When FeCl_2_ or CuCl_2_ were added to individual solutions containing **APH1**–**APH5**, and when HgCl_2_ was added to solutions containing **APH3**, **APH4**, or **APH**5, new optical bands did not appear, and, therefore, the wavelengths corresponding to the maximum absorption bands of the Fe^2+^/compound, Cu^2+^/compound, or Hg^2+^/compound solutions were similar to those of the compounds alone. By contrast, the analysis of solutions at different Fe^2+^/compound, Cu^2+^/compound, and Hg^2+^/compound molar ratios evidenced important differences regarding the intensity of the absorption bands. The observed changes suggest an interaction between the tested compounds and Fe^2+^, Cu^2+^, and Hg^2+^ and, consequently, the formation of iron, copper, and mercury complexes, respectively. However, when the same experiments were performed with ZnCl_2_, CdCl_2_, or AlCl_3_ and solutions containing **APH1**–**APH5**, and when HgCl_2_ was added to solutions containing **APH1** or **APH2**, no differences between spectra of the different Zn^2+^/compound, Cd^2+^/compound, Al^3+^/compound, and Hg^2+^/compound molar ratios and the compounds alone were observed. This absence of spectral changes suggests the lack of interaction between the tested compounds and Zn^2+^, Cd^2+^, Al^3+^, and Hg^2+^.

Due to the high spectral similarity between the compounds and their Fe^2+^, Cu^2+^, Hg^2+^, Zn^2+^, Cd^2+^, or Al^3+^ complexes, derivative spectroscopy was applied in order to enhance differences among spectra, to resolve overlapping bands, to reduce the effects of other absorbing compounds, and to eliminate the background. Once UV–vis spectra were registered, mathematical methods were used to generate the first-order derivative spectra. Our analysis shows excellent results in enhancing differences among spectra, increasing the resolution of the overlapped bands, reducing the effects of other absorbing compounds, and eliminating the background. In fact, as the first derivative spectrum passes through zero at the same wavelength as λ_max_ of the absorbance band, the spectral differences are highlighted. Thus, the analysis of derivative spectra allowed to confirm, through a resolution enhancement effect, that **APH1**–**APH5** were able to interact with Fe^2+^ and Cu^2+^. Moreover, it was verified that **APH3**, **APH4**, and **APH5** were also able to interact with Hg^2+^ and, thus, to form iron, copper or mercury complexes. Otherwise, no changes were appreciated in Hg^2+^-**APH1** and Hg^2+^-**APH2** and neither in Zn^2+^, Cd^2+^, or Al^3+^with **APH1**–**APH5** from derivative spectra.

**Figure 2:**
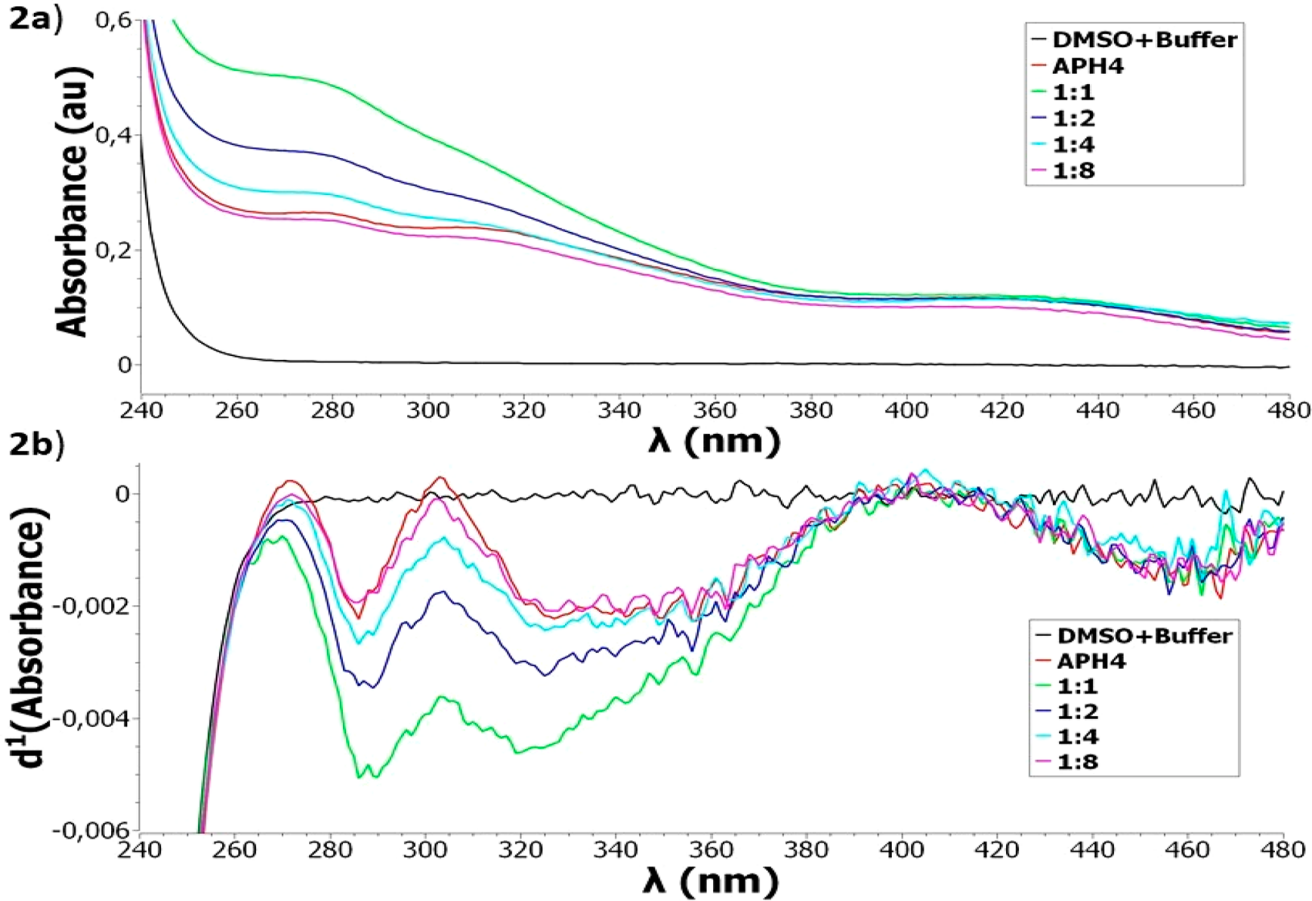
UV–vis spectra (a) and first-order derivative absorption spectra (b) of **APH4** alone and in the presence of FeCl_2_ in buffer (100 mM phosphate, pH= 7.30) at room temperature. [**APH4**] = 50 μM and [Fe^2+^] = 50, 25, 12.5, and 6.25 μM, corresponding to stoichiometries 1:1, 1:2, 1:4 and 1:8, respectively.

The stoichiometry of the chelated iron, copper, and mercury compounds were estimated by means of the derivative spectra collected under two different conditions: (i) maintaining constant the metal ion concentration (50 μM) and varying the compound concentration and (ii) maintaining constant the compound concentration (50 μM) and varying the metal concentration. The results showed that the five compounds evaluated presented a similar behavior regarding Fe^2+^ and Cu^2+^ chelation. Figure 2a,b shows the UV–vis spectra registered for Fe^2+^-**APH4**, as an example of metal-compound interaction. Figure 2a evidenced differences between absorbance spectra of the compound alone and in the presence of FeCl_2_. Nevertheless, differences among the spectra corresponding to different stoichiometries are not properly appreciated. However, as depicted in Figure 2b, first-order derivative absorption spectra revealed some differences depending on the evaluated stoichiometry. The maximum absorption band at 280 nm (Figure 2a) appeared in all stoichiometries, being associated with the negative band at 290 nm as reflected in the derivative spectra (Figure 2b). This band may correspond to the absorption of the iron complex as it increases, as metal concentration reaches its maximum in Figure 2a. Otherwise, a ligand absorption band was observed at 300 nm, which overlaps with the optical band of the Fe^2+^ complex. This effect is shown in Figure 2b, where the derivative spectra of the compound alone passed through 0 at 300 nm wavelength, in contrast with the different stoichiometries. By analysis of the first-order derivative spectra (Figure 2b), the stoichiometry of the Fe^2+^-**APH4** complex is consistent with a 1:1 Fe^2+^/compound molar ratio. However, complexes of different stoichiometry could be simultaneously formed.

Similar observations were made for Fe^2+^ and Cu^2+^ with **APH1**–**APH5** and for Hg^2+^ with **APH4** and **APH5**. The spectra revealed bands that could be associated with the iron complexes at wavelengths of 280 nm (Fe^2+^-**APH1**), 270 and 305 nm (Fe^2+^-**APH2**), 280 nm (Fe^2+^-**APH3**), and 270 nm (Fe^2+^-**APH5**), while the copper complexes showed bands at 275 and 300 nm (Cu^2+^-**APH1**), 265 nm (Cu^2+^-**APH2**), 260 nm (Cu^2+^-**APH3**), 265 nm (Cu^2+^-**APH4**), and 270 nm (Cu^2+^-**APH5**). Moreover, with the mercury complexes bands appeared at 295 and 300 nm (Hg^2+^-**APH4**) and 300 and 305 nm (Hg^2+^-**APH5**). Once again the 1:1 metal/compound molar ratio seems to be the main common stoichiometry for the formed complexes. However, for Hg^2+^-**APH3**, showing an absorption band at 305 nm, the stoichiometry remains unclear.

**Figure 3:**
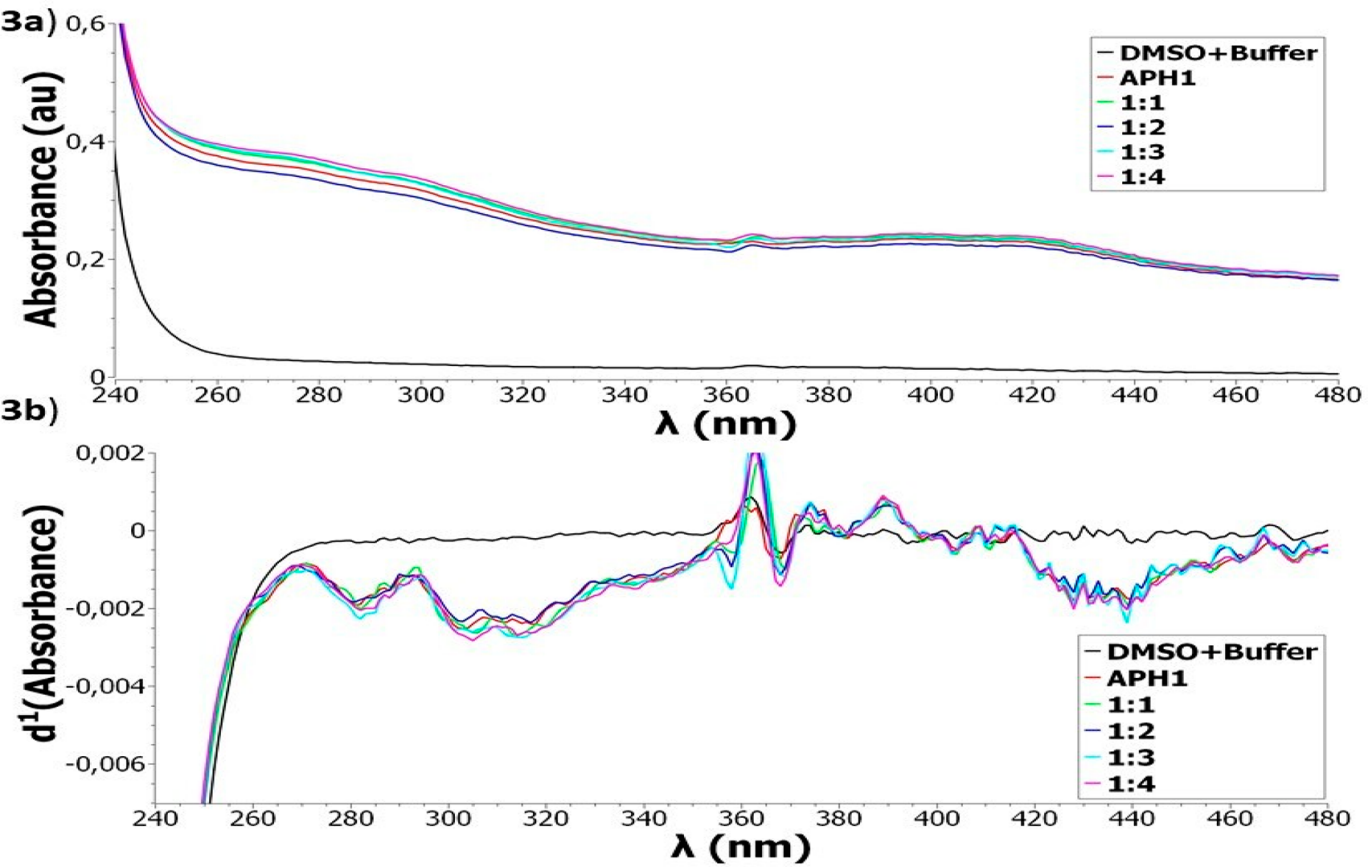
UV–vis spectra (a) and first-order derivative absorption spectra (b) of **APH1** alone and in the presence of CdCl_2_ in buffer (100 mM phosphate, pH= 7.30) at room temperature. [**APH1**] = 50 μM and [Cd^2+^] = 50, 25, 16.7, and 12.5 μM corresponding to stoichiometries 1:1, 1:2, 1:3 and 1:4, respectively.

Figure 3a,b shows the Cd^2+^-**APH1** spectra, as an example of an noninteracting metal-compound. There were no differences between compound bands (**APH1**) and metal-compound bands at different molar ratios (1:1–1:4) in the UV–vis spectra (Figure 3a). These differences were not appreciated in the first derivative spectra (Figure 3b). Similar observations were deduced for Zn^2+^, Cd^2+^ and Al^3+^ with **APH1**-**APH5** and for Hg^2+^ with **APH1** and **APH2**. The rest of UV–vis and first-order derivative absorption spectra data for **APH** compounds and the metals studies are shown in Figures S3–S7 in the Supporting Information.

In our previous work, we showed that **APHs** are able to inhibit MMP enzymes and computational studies suggested that this inhibitory activity is not mediated by the chelation of Zn^2+^ ion at the catalytic site, as is the case for many other MMP inhibitors.^17^ Our present results show that **APH** compounds tested were not able to chelate Zn^2+^ ion, confirming the computational results. Further analysis should be performed to determine the mode of action behind MMPs inhibition by **APHs**.

### Highest Occupied Molecular Orbital and Lowest Unoccupied Molecular Orbital analysis

The obtained results also show that all five **APH** compounds are able to bind to Fe^2+^ and Cu^2+^, while they are unable to bind to bigger cations such as Cd^2+^, Al^3+^, and Hg^2+^. The Hg^2+^ binding capacity of **APH3**–**APH5** is probably due to coordination with the nitrogen atom of dimethylamino group for **APH3** and with the thiophen sulfur of **APH4** and **APH5**. Furthermore, Zn^2+^ and Cd^2+^ ions usually form five-membered complexes with bidentate species, while Fe^2+^ and Cu^2+^ preferentially lead to the formation of six-membered metallocycles, consistent with the observed results.

To corroborate our experimental results of the chelating capacity of **APH** compounds and to relate these results with the compound structures, we studied highest occupied molecular orbital (HOMO) and lowest unoccupied molecular orbital (LUMO) orbital energies and energy gaps for the **APH** compounds and for the corresponding **APH-Fe** complexes (Table S1). Iron was chosen since, together with copper, it was chelated by all APH compounds. Our results for compounds **APH1**–**APH5** show that the proposed metal binding site was accessible (without impediment from the substituent of the exocyclic nitrogen in the most stable conformation calculated) for the five compounds (Figure S1). It was also observed that the HOMO orbital is mainly extended on the alkyl or the π system at the C-7 position. Particularly, for **APH3**, the charge density is concentrated on the dimethylamino group. Finally, the HOMO–LUMO energy gap (7.049, 7.799, 4.494, 5.982, and 6.643 eV for **APH1**–**APH5**, respectively), calculated at Merck molecular force field 94 (MMFF94) level, is compatible with complex formation.

**Figure 4:**
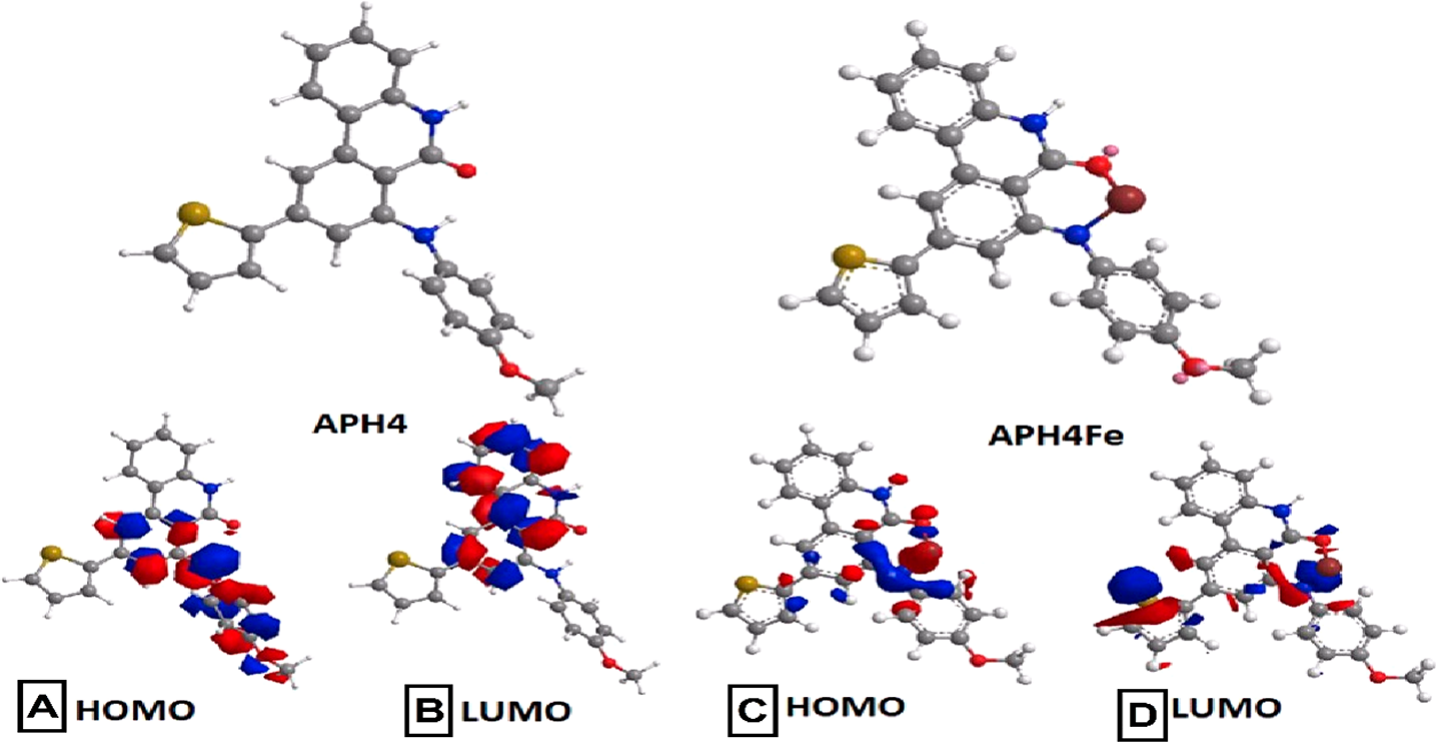
Optimized molecular structure of the **APH4** and **APH4-Fe** complex (upper) and their corresponding HOMOs (A and C) and LUMOs (B and D). Color scheme: Carbon is gray, hydrogen is white, nitrogen is blue, oxygen is red, and iron is brown. HOMOs and LUMOs are shown in red and blue.

The HOMO and LUMO levels for the **APH** compounds complexed with iron showed a reduction of gap energy of 0.917, 0.712, 3.922, 0.145, 0.299 eV for **APH1-Fe** to **APH5-Fe** complexes, respectively (Table S1), indicating a high stability for the complexes. The highest reduction of gap energy was observed in **AHP4-Fe**, showing that this compound is the most stable (Table S1) and therefore that **AHP4** has the highest chelating activity, a prediction that correlates well with the fact that this compound presented the best neuroprotective activity as shown below. Taking **APH4** as an example, it is apparent that the exocyclic nitrogen gives a higher contribution to the HOMO levels of **APH4** than to the HOMO levels of the **APH4-Fe** complex (Figure 4a,c), which indicates that the nitrogen atom depletes charge from the Fe^2+^ ions. Additionally, the presence of an aromatic amine at C-7 seems to stabilize the iron complex in comparison to the alkyl amine (see **APH1-Fe** and **APH2-Fe** in Table S1), and the amine group on the aromatic ring seems to destabilize the complex compared to the methoxy group (see **APH3-Fe** in Table S1). Finally, the presence of a thiophene at the C-9 position appears to lead to a smaller gap than the presence of a benzene ring (compare **APH4-Fe** and **APH2-Fe** in Table S1).

Therefore, although the number of compounds studied is somewhat limited to conclude a structure-complex formation ability relationship, it could be confirmed that their ability to interact with Fe^2+^ and Cu^2+^ does not depend on the substituent of 7-amino phenanthridi-6-one skeleton. The computational analysis also revealed that phenyl moieties on the exocyclic nitrogen by the iron complex stabilizes the HOMO charge. Thus, the chelating cavity is suitable for small metal cations.

### Computational Prediction of Physicochemical and Absorption, Distribution, Metabolism, Excretion, and Toxicological Properties of **APH** Compounds

According to the ADMETsar version 2.0 and swissADME software analysis, as shown in Table 1, the **APH** compounds satisfied all the values required except for the lipophilicity values,^35^ which are slightly above the Lipinski limit.

**Table 1:** Predicted Physicochemical Properties of **APH** Compounds

property	**APH1**	**APH2**	**APH3**	**APH4**	**APH5**	favorable value
Drug-likeness						
molecular weight (g/mol)	342.44	392.46	405.50	398.49	368.46	≤500
log *P*	5.56^a^; 5.03^b^	6.10^a^; 5.16^b^	6.16^a^; 5.18^b^	6.19^a^; 5.21^b^	6.15^a^; 5.31^b^	≤5
no. rotatable bonds	5	4	4	4	3	≤7
no. H-bond acceptors	2	3	3	2	3	≤10
no. H-bond donors	2	2	2	2	2	≤5

a Prediction with ADMETsar version 2.

b Prediction with Swiss ADME.

Based on the results shown in Table S2, it can be assumed that **APH** compounds have favorable intestinal absorption and Caco2 cell permeability. The CNS distribution properties of our molecules was estimated through the calculation of the brain distribution parameter, which can evaluate the ability of small molecules to cross the blood–brain barrier (BBB) and be distributed into the brain. The predictions of the two methods used were fully consistent for **APH1**, where both predicted a good BBB penetration ability. For **APH2**, **APH3**, **APH4** and **APH5**, ADMETsar version 2.0 showed good BBB penetration data, but swissADME gave borderline negative results. The metabolic properties of these compounds were estimated according to the predicted inhibitory capacity of cytochrome P450 isoforms Cyp1a2, Cyp2c19, Cyp2c9, and Cyp2d6. As an additional piece of information connected to the prediction of pharmacokinetic properties, glycoprotein substrate capacity was assessed, but again the results from the two softwares were not homogeneous. To summarize, it was found that, although the lipophilicity scores may need to be further optimized, the predicted absorption, distribution, metabolism, excretion, and toxicological (ADMET) properties of the **APH** compounds characterize them as a suitable starting point to develop a leader compound. All the predicted characteristics of **APHs** in passing the BBB, permeability to Caco-2 cells, interaction of the compound with the cytochrome P450 isoenzymes and P-glycoproteins are shown in Table S2.

### Neuroprotective Activity of Compounds **APH1**–**APH5** against Metal-Induced Cell Viability Reduction

**APH1**-**APH5** were selected because they were, out of all the **APH** compounds tested, the ones with less cytotoxic effects, also presenting the most potent antioxidant and neuroprotective effect against ROS isults.^17^ Thus, the treatment of SN56 cells with **APH1**–**APH5** at different concentrations (1, 10, 50, 100, 150, and 200 μM) only started to reduce cell viability at 200 μM (Figure S2). Thus, the range of 10–100 μM concentrations was selected, as it has shown no cytotoxicity, to tests **APHs** neuroprotective effects.

Treatment of neurons with the heavy (cadmium, mercury, and aluminum) and essential (iron, copper, and zinc) metals’ produced a reduction of cell viability. This effect was avoided completely or partially when cells were pretreated with **APH1**–**APH5** in a concentration-dependent way (Figures 3 and 4). The heavy and essential metals studied were reported to induce neuronal cell death,^1−8^ supporting our results.

**Figure 5:**
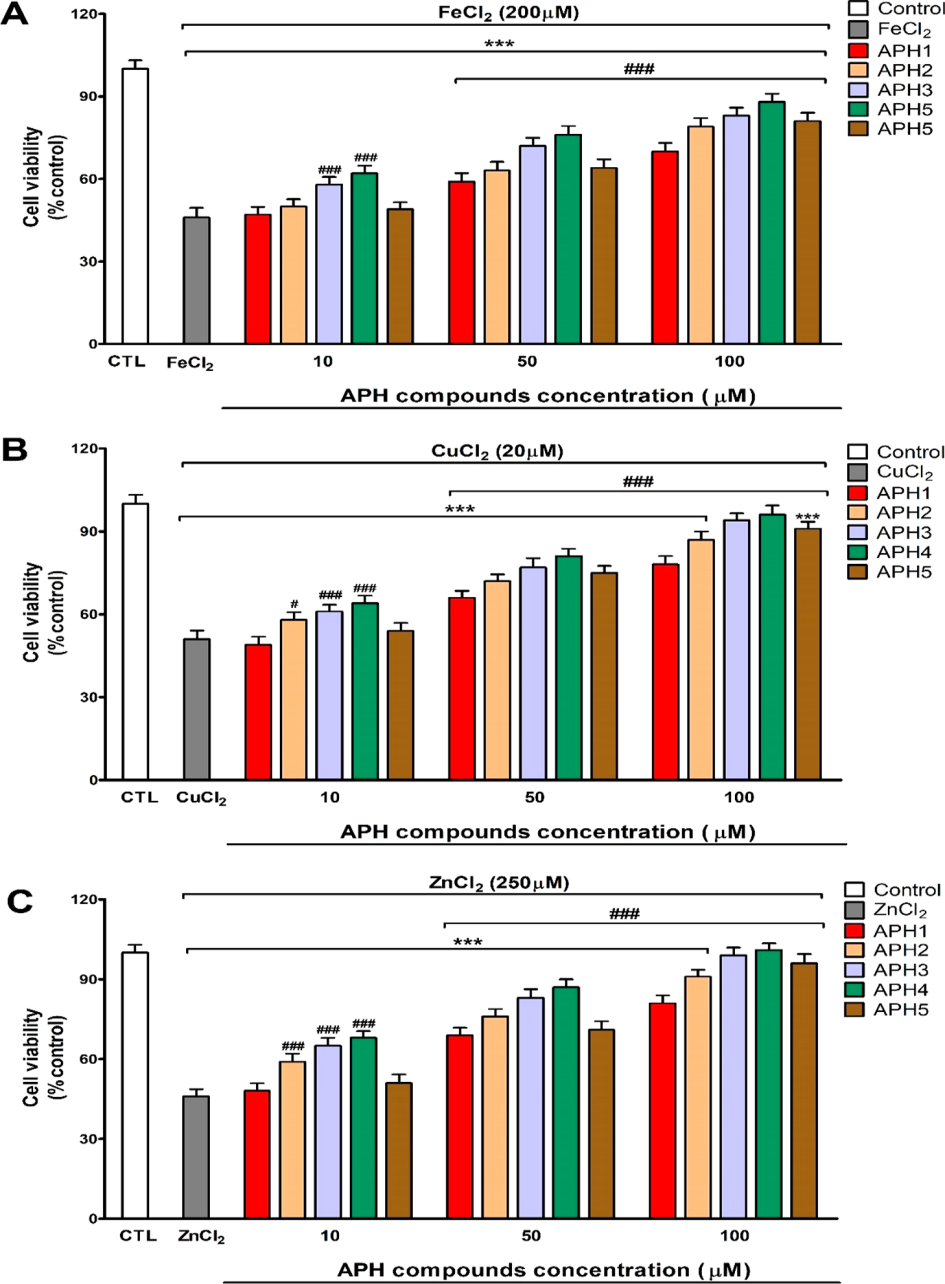
Cell viability effects after FeCl_2_ (A) for CuCl_2_ (B) and ZnCl_2_ (C) treatment with or without selected **APH** compounds (10–100 μM) pretreatment. Data represent the mean ± SEM of three independent experiments in triplicate. ***p ≤ 0.001 compared to control; ^#^*p* ≤ 0.05 and ^###^*p* ≤ 0.001 compared to metal treatment.

**Figure 6:**
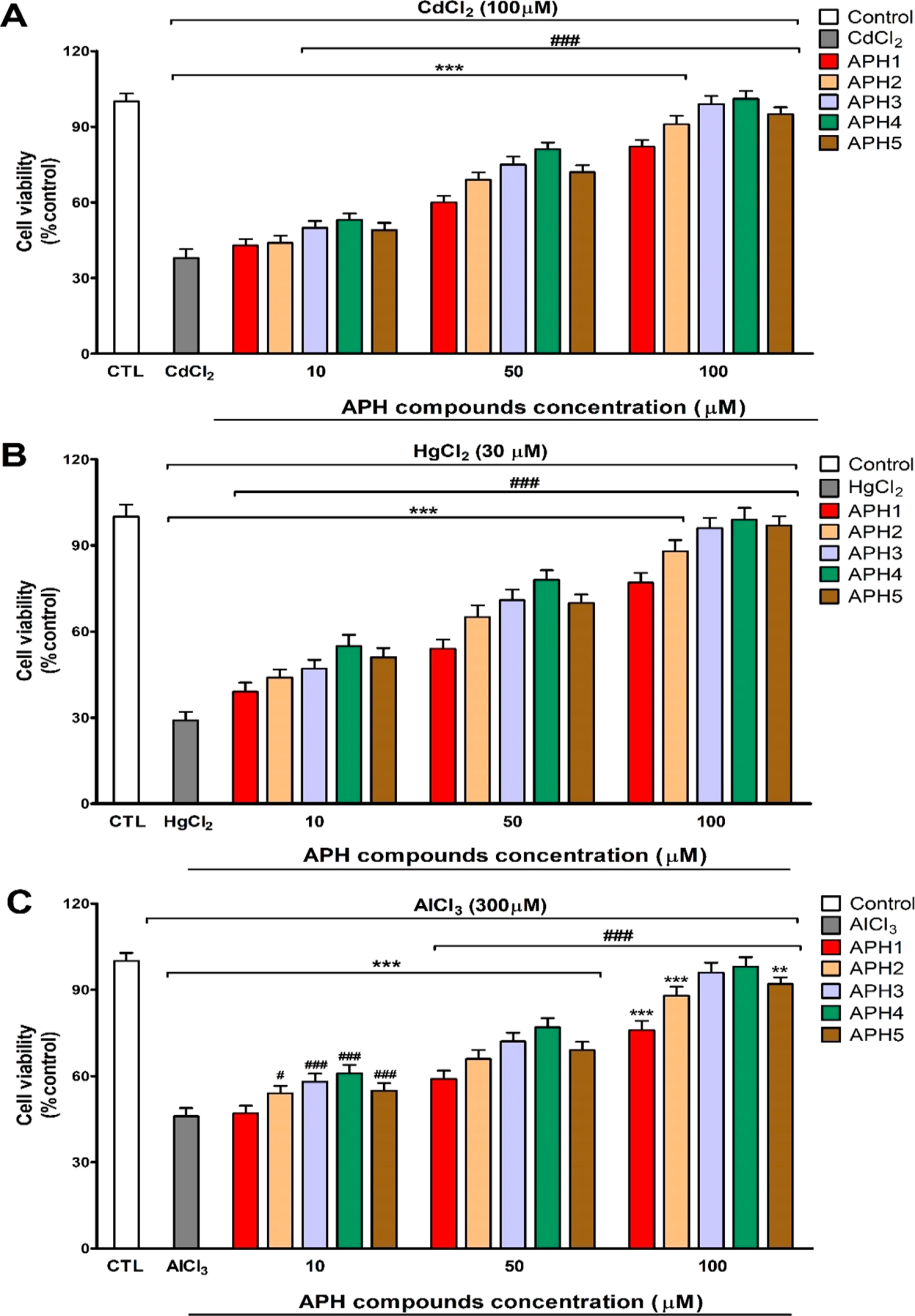
Cell viability effects after CdCl_2_ (A) for HgCl_2_ (B) and AlCl_3_ (C) treatment with or without selected **APH** compounds (10–100 μM) pretreatment. Data represent the mean ± SEM of three independent experiments in triplicate. **p ≤ 0.01 and ***p ≤ 0.001 compared to control; *p* ≤ 0.05 and ^###^*p* ≤ 0.001 compared to metal treatment.

The protection against essential metals started from 10 μM after pretreatment with **AHP2**, **APH3**, or **APH4**. For FeCl_2_, pretreatment with **APH2** started the protection from 50 μM as well as after the pretreatment with **APH1** or **AHP2** in cells treated with essential metals (Figure 5a–c). Besides, in cells treated with heavy metals, the **APH1**–**APH5** pretreatment started to protect, from 10 μM, against the cell viability reduction observed after heavy metals treatment alone. For CdCl_2_, pretreatment with **APH1** and **APH2** and for AlCl_3_, pretreatment with **APH1** started cell death reversion from 50 μM (Figure 5a–c). Pretreatment with compounds **APH3** and **APH4**, at the concentration of 100 μM, protected completely against the cytotoxic effects induced by all metals studied except for FeCl_2_ (Figures 5 and 6). Besides, pretreatment with compound **APH5**, at the concentration of 100 μM, only protected completely against the cytotoxic effects induced by ZnCl_2_, CdCl_2_, and HgCl_2_ (Figures 5 and 6).

The differences observed in the concentrations from which these compounds start to show protection against the metal-induced cytotoxic effects can be ascribed to the different potency in their different protective mechanisms. These differences may also be produced because each APH compound may induce different mechanisms that mediate the neuroprotection observed. In this sense, our chelating studies suggest that Zn^2+^, Cd^2+^, and Al^3+^ ions are not chelated by any of the **APH** compounds studied and that Hg^2+^ is only chelated by **APH3**, **APH4**, and **APH5** compounds. This fact indicates that the neuroprotection observed against zinc, cadmium, and aluminum by all these compounds or against mercury by compounds **APH1** and **APH2** is not mediated by their chelating activity and other mechanisms are involved. Besides, the protection observed against iron, and probably copper, at the lowest compound concentrations should be limited, especially for iron. The chelating activity at the higher concentrations used of these metals only blocked a very limited part of the metal pool, suggesting other mechanisms are involved. In this sense, we previously described that these compounds are MMP inhibitors and present antioxidant activity,^17^ which could participate in these neuroprotective effects. The greater neuroprotective action observed after pretreatment with all metals studied was mediated by compound **APH4**. This effect is correlated with our previous results on MMPs inhibition and antioxidant effect^17^ that support that these mechanisms are probably involved in the neuroprotective effects of these compounds.

### NRF2 Pathway Induction by Compounds **APH1**–**APH5**

**Figure 7:**
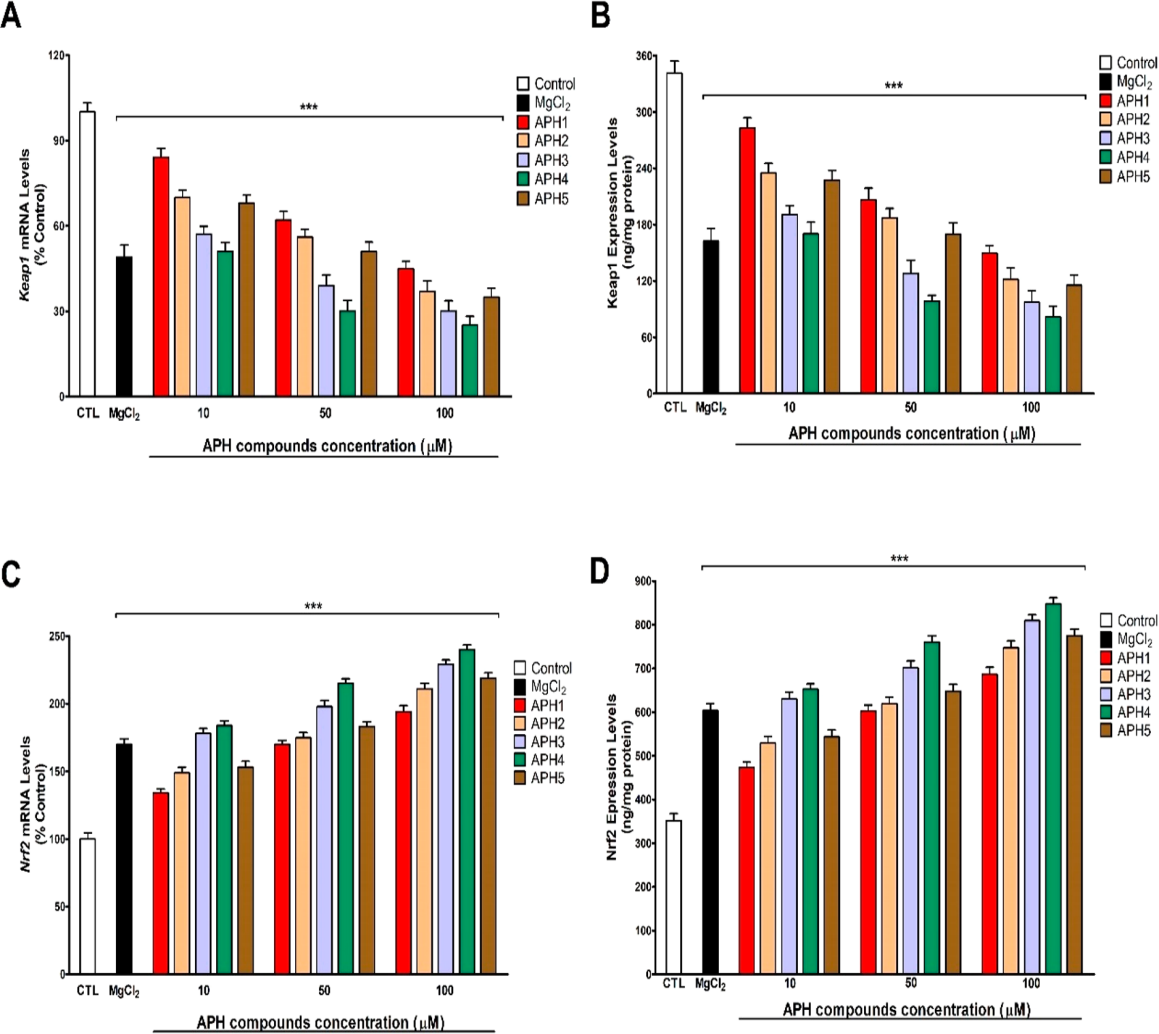
Obtained results from *Keap1* (A) and *Nrf2* (C) gene expression and Keap1 (C) and Nrf2 (D) protein expression after 24 h treatment. *Keap1* and *Nrf2* gene and protein expressions are compared with controls [cells treated with vehicle were the negative control]. Each bar represents mean ± SEM of three separate experiments from cells of different cultures, each one performed in triplicate and presented as percent untreated control for gene expression and in ng/mg protein form protein expression. ***p < 0.001 significantly different from controls.

**Figure 8:**
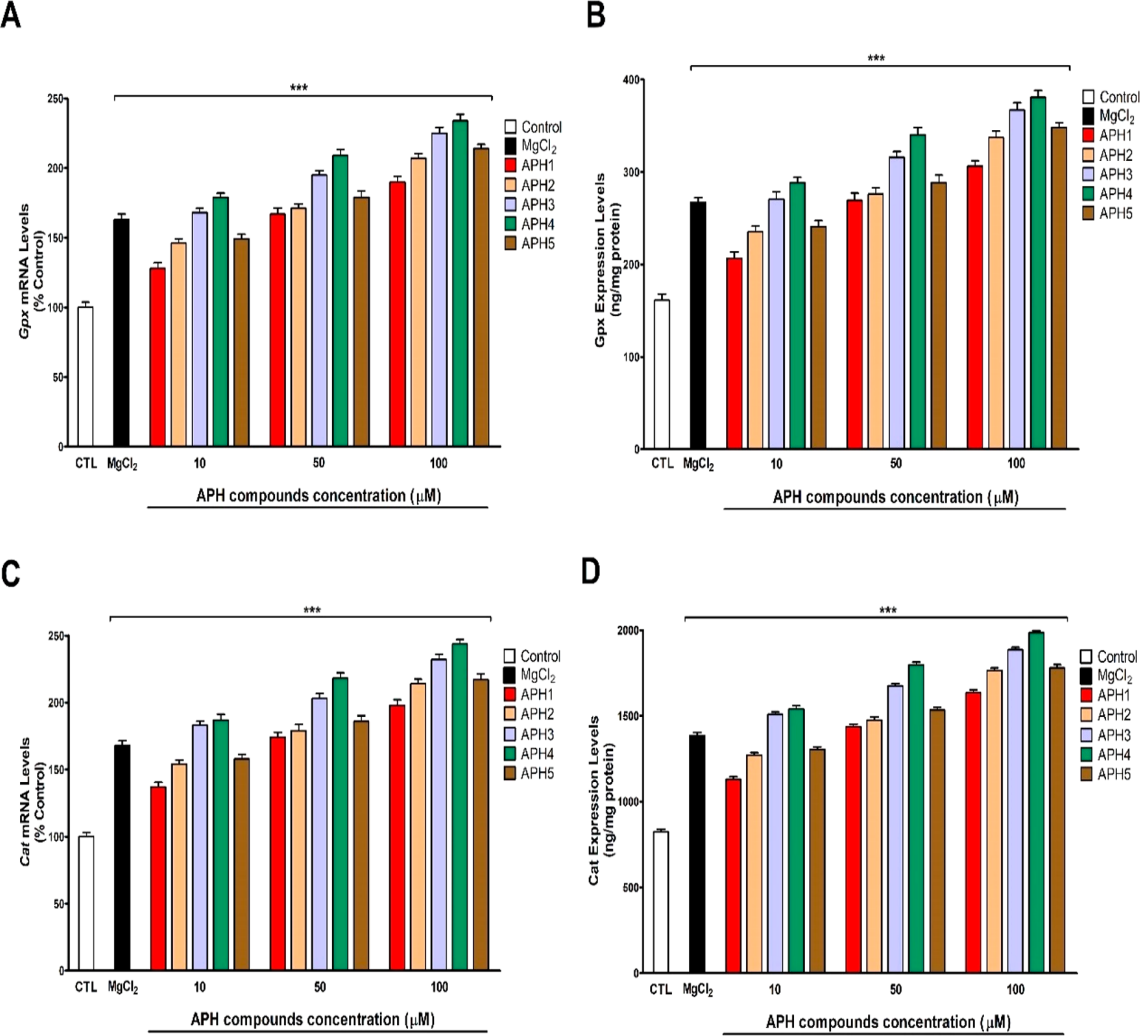
Obtained results from *Gpx* (A) and *Cat* (B) gene expression and Gpx (C) and Cat (D) protein expression after 24 h treatment. *Gpx* and *Cat* gene and protein expressions are compared with controls [cells treated with vehicle were the negative control]. Each bar represents mean ± SEM of three separate experiments from cells of different cultures, each one performed in triplicate and presented as percent untreated control for gene expression and in ng/mg protein for protein expression. ***p < 0.001 significantly different from controls.

The treatment of SN56 cells with compounds **APH1**–**APH5** induced a concentration-dependent decrease in the gene and protein expression of Keap1 (Figure 7a,b). A correlated concentration-dependent increase in the gene and proteins expression of Nrf2 factor (Figure 7c,d) was observed. Besides, the gene and protein expression of Gpx and Cat enzymes was increased, in a concentration-dependent way, after the treatment with compounds **APH1**–**APH5** (Figures 8a–c). Thus, **APH** compounds induce Nrf2 pathway.

Keap1 protein regulate Nrf2 proteins levels, inducing its degradation under no stress situations, through proteasome degradation. However, after stress situations, like ROS generation, its affinity for Nrf2 protein is reduced, increasing Nrf2 levels. Nrf2 is then translocated to the nucleus where it acts as the master regulator of the expression of different antioxidant enzymes such as Gpx and Cat among others.^18−20,36,37^*Keap1* downregulation was reported to induce Nrf2 protein overexpression and the enzymes regulated by it.^38,39^ Besides, *Keap1* and *Nrf2* gene expression was reported to be regulated by different factors like microRNAs (miRNAs) or different transcription factors.^38,40^ Nrf2 protein levels were shown to be altered by mechanisms independent of Keap1 protein action.^36^ In this sense, we showed that compounds **APH1**–**APH5** induced the Nrf2 pathway by different mechanisms. On one hand, they induce the Nrf2 pathway by an independent mechanism of Keap1 regulation of Nrf2 proteins degradation, since these compounds act to upregulate the *Nrf2* gene expression. On the other hand, compounds **APH1**–**APH5** regulate the Nrf2 pathway by a mechanism dependent on keap1, since they downregulated *Keap1* gene expression, contributing to the overexpression of Nrf2 protein and of the Gpx and Cat antioxidant enzymes regulated by it. Further studies are needed to determine the mechanism through which **APH** compounds mediate the *Keap1* and *Nrf2* gene expression alteration.

We previously described that **APH** compounds present antioxidant activity against ROS-induced lipid peroxidation.^17^ In the present study, we show that this action is mediated through the induction of Nrf2 pathway, but we cannot discard other mechanisms are involved. Besides, the greater effect on Keap1, Nrf2, Gpx, and Cat expression was mediated by compound **APH4** (Figures 7 and 8), which is correlated with the higher antioxidant effect observed for this compound,^17^ supporting our results.

### Antioxidant Activity of Compounds **APH1**–**APH5** against Metal-Induced Lipid Peroxidation

**Figure 9:**
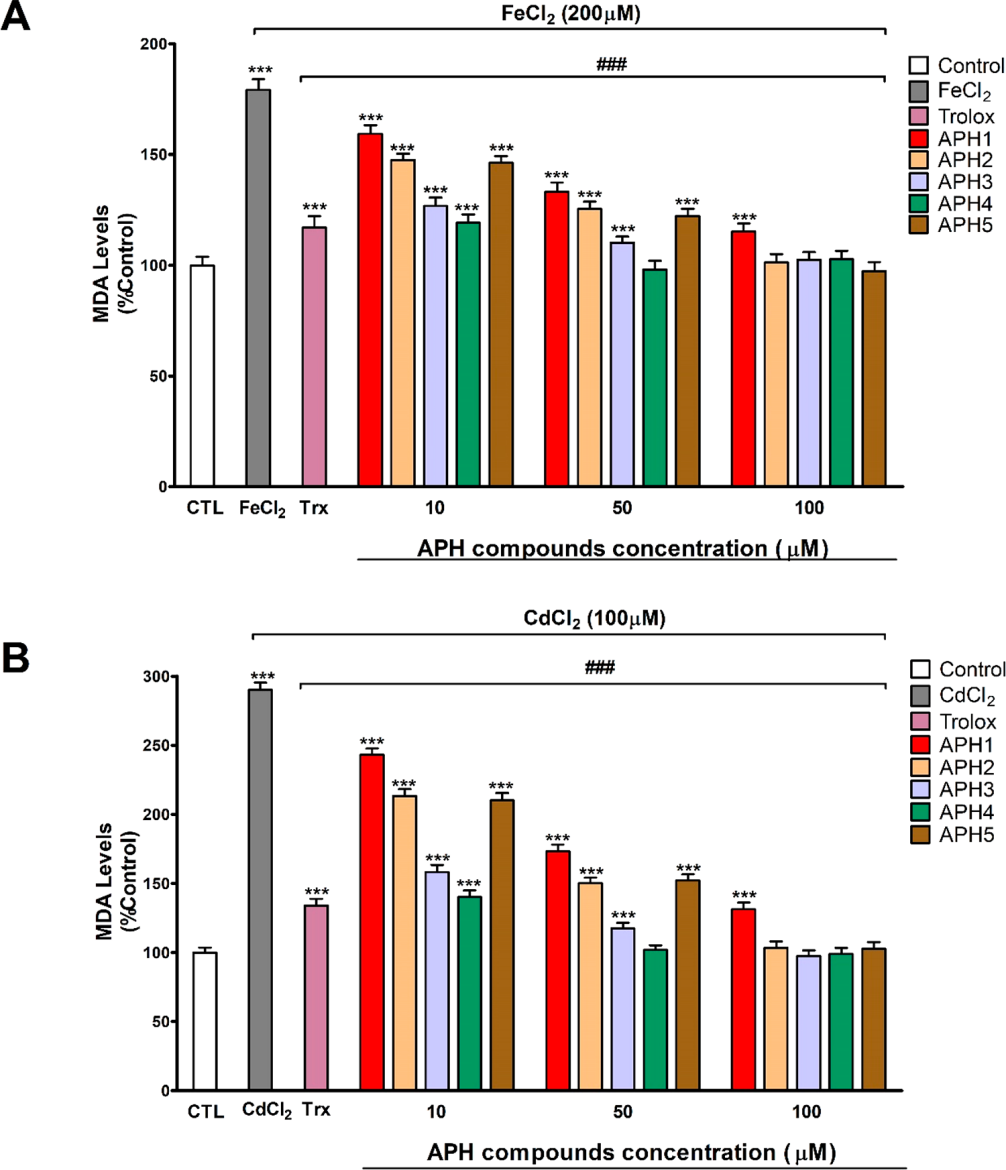
Neuroprotective action of selected **APH** compounds against lipid peroxidation induced by FeCl_2_ and CdCl_2_ metals in SN56 cells was measured by MDA assay. (A) MDA content after FeCl_2_ treatment with or without **APH** compounds. (B) MDA content after CdCl_2_ treatment with or without **APH** compounds. Values are given as mean ± SEM of three separate experiments from cells of different cultures, each one performed in triplicate and presented as percent untreated control. ***p ≤ 0.001 compared to control; ^###^*p* ≤ 0.001 compared to metal treatment.

The heavy (CdCl_2_) and essential (FeCl_2_) metals generation of malondialdehyde (MDA) was determined as a marker of oxidative stress damage induced by metals. MDA, a product of the ROS-promoted degradation of arachidonic acid, is the best-known lipid peroxidation marker. Obtained results show that both metals induced lipid peroxidation at the concentrations used (Figure 9a,b). Concentrations used were selected because they were the ones that produce cell damage (data no shown). Several *in vitro* studies reported that FeCl_2_ and CdCl_2_ induced, at these concentrations, oxidative stress and cell death,^8,41,42^ supporting our results.

Treatment of SN56 cells with **APH** compounds alone did not produce any effect on MDA levels (data not shown). Pretreatment with the antioxidant trolox at 100 μM concentration prevented partially the lipid peroxidation induced by cadmium and iron (Figure 9a,b). Pretreatment with compounds **APH1**, **AHP2**, **APH3**, **APH4**, or **APH5**, prior to heavy or essential metal treatment, averted lipid peroxidation in a concentration-dependent way, starting from the 10 μM concentration (Figure 9a,b). This protection was complete after pretreatment with **APH4** compound at 50 μM concentration in both metals (Figure 9a,b). Finally, pretreatment with compounds **APH2**, **APH3**, or **APH5**, at 100 μM concentration, the same one used for trolox, protected completely against the lipid peroxidation induced by both metals (Figure 9a,b). Thus, compounds **APH2**–**APH5** are more potent than trolox. These data support the antioxidant effect of these compounds not only after H_2_O_2_ insults as previously shown^17^ but also against all insults induced by ROS generated after metals exposure.

Otherwise, the compound **APH4** was not able to protect completely against the cell damage induced by any metal at the concentration of 50 μM (Figures 5 and 6). However, it was able to avoid completely the oxidative stress induced by FeCl_2_ and CdCl_2_ (Figure 9a,b). Besides, the compounds **APH3**, **APH4**, and **APH5**, at the concentration of 100 μM, were not able to completely protect against the cell damage induced by FeCl_2_ (Figure 5a), but they prevented completely the oxidative stress induced by this metal (Figure 9a). These facts indicate that, besides the oxidative stress, additional mechanisms should be involved in the cytotoxicity of these metals. It also suggests that, in addition to the antioxidant effect of these compounds, they mediate their neuroprotective effect by other mechanisms, probably different than MMPs inhibition or the chelating activity depending of the metal. These metals were reported to induce the accumulation and aggregation of amyloid proteins leading to cell death.^1−3^ Moreover, these metals were related with the alteration of Wnt/β-catenin pathway and glutamatergic system and the induction of neuroinflammation, producing neurodegeneration.^1−3,43,44^ Studies to elucidate the neuroprotective mechanisms of **APH** compounds, besides of those commented already, have not been previously performed. However, the parent compound 6(5H)-phenanthridinone was described to present poly(ADP-ribose)polymerase (PARP) inhibitor activity.^45^ PARP was involved in inflammation induction. Its inhibition produces anti-inflammatory effects,^46^ and it was also observed that its inhibition increases the NRF2 nuclear and cytosolic levels in rat retinal cells injured by chronic hypoxia/reoxygenation process.^47^ Thus, **AHP1**–**APH5** compounds could mediate their antioxidant effects as well as other neuroprotective mechanisms through PARP inhibition. Besides, phenanthridin-6(5H)-one derivatives were also shown to act as Wnt/β-catenin signaling pathway agonists.^48^ Additionally, Wnt/β-catenin signaling pathway activation was reported to possibly induce the NFR2 expression through GSK-3β inhibition.^49^ Therefore, **APH** compounds could also block these cytotoxic actions through these mechanisms, contributing together with the antioxidant, chelating, and MMPs inhibition activities to the neuroprotective effects observed against the neurotoxic action of these metals. Further studies are required to determine the rest of the neuroprotective mechanisms of these multitarget compounds that could be used in the treatment of different neurodegenerative diseases.

## Conclusions

To summarize, all compounds **APH1**–**APH5** were able to chelate iron and copper ions. Moreover, **APH3**, **APH4**, and **APH5** were also able to chelate mercury ions. Nevertheless, none of the **APH** compounds was able to chelate zinc, cadmium, and aluminum. The cytotoxic effects induced by all studied metals were prevented completely or partially depending on the compound used, with the most promising compound **APH4**. However, it is necessary to corroborate the neuroprotection action *in vivo* and against other toxic stimuli to confirm **APH4** compound as the best **APH** therapeutic tool.

Otherwise, the antioxidant effect of the evaluated compounds is mediated through induction of the Nrf2 pathway that leads to overexpression of antioxidant enzymes. Further studies are required to survey the rest of the possible mechanisms involved in the neuroprotective effect of these compounds.

Consequently, the relevance of our results lies in finding the chelating, antioxidant, and neuroprotective properties of new multitarget 7-aminophenanthridin-6(*5H*)-one derivatives that could help improve the treatment management of different neurodegenerative diseases.

## Methods

### General Experimental Information

All reagents were purchased from Sigma-Aldrich and Fluka (Madrid, Spain), and solvents from SDS (Madrid, Spain) were of commercial quality and were used as received. Reactions were monitored by thin-layer chromatography, 0.20 mm silica gel 60 F254 plates (Merck, Madrid, Spain) with fluorescent indicator (SDS CCM221254), and then visualized with an ultraviolet (UV) lamp. Separations by flash chromatography were performed on silica gel (SDS 60 ACC 15 40–63 μm). Infrared spectra were recorded on a PerkinElmer Paragon 1000 FT-IR spectrophotometer (PerkinElmer, Madrid, Spain). NMR spectra were obtained on a Bruker Avance 250 spectrometer working at 250 MHz for ^1^H and 63 MHz for ^13^C and operated via the standard Bruker software (Nuclear Magnetic Resonance, Centre for Research Assistance, Complutense University, Madrid, Spain). Chemical shifts are reported in parts per millions relative to tetramethylsilane, and spin multiplicities are given as s (singlet), d (doublet), t (triplet), q (quartet), or m (multiplet).

### General Synthesis Procedure for the Target **APH** Compounds

#### Synthesis of **1**

A solution of ethyl acetoacetate (3 mmol, 1 equiv) and the suitable primary amine (3–3.9 mmol, 1–1.3 equiv) in ethanol (5 mL) was added to cerium(IV) ammonium nitrate (5 mol) and was stirred for 30 min at room temperature. Then, appropriate *ortho*-nitrochalcone (2.4–3.6 mmol, 0.8–1.2 equiv) was added to the stirred solution, and the mixture was heated to 80 °C overnight. The reaction mixture was dissolved in ethyl acetate (30 mL) and washed consecutively with water and brine, and the organic layer was dried with anhydrous Na_2_SO_4_. The solvent was removed under reduced pressure, and the solid was purified by flash column chromatography on silica gel, eluting with a petroleum ether and ethyl acetate mixture (9:1, v/v). Compounds **1** were reported in the literature, and their characterization data were identical to those previously described.^17^

#### Synthesis of **2**

A solution of appropriate dihydroantranilate **1** (1.5 mmol) and DDQ (1.5 equiv) in 10 mL of toluene was stirred (10 mL) for 2 h at room temperature. Then, the reaction mixture was extracted with ethyl acetate (30 mL × 3), and the combined organic phase was washed with 30 mL water and 30 mL brine solution. The residue was concentrated under vacuum, and the crude was purified by flash chromatography on silica gel with a petroleum ether and ethyl acetate gradient (from 9/1 to 8/2). Compounds **2** were reported in the literature, and their characterization data were identical to those previously described.^17^

#### Synthesis of **APH**

A solution of appropriate *meta*-terphenyl compound **2** (1 mmol) was dissolved in ethanol (25 mL), and 10% palladium on carbon (Pd/C) (0.1 mmol, 10 mol %) was added. The suspension was stirred at room temperature for 14 h under hydrogen atmosphere, the catalyst was removed by filtration over Celite and washed with dichloromethane, and the solvent evaporated. The crude was purified by column chromatography on silica gel, eluting with a petroleum ether-ethyl acetate gradient (from 8:2 to 1:1). Compounds **APH** were reported in the literature, and their characterization data were identical to those previously described.^17^

##### 7-(Butylamino)-9-phenylphenanthridin-6(*5H*)-one (**APH1**)

^1^H NMR (250 MHz, DMSO-*d*_6_) δ 9.46 (t, *J* = 4.9 Hz, 1H), 8.42 (d, *J* = 7.9 Hz, 1H), 7.86 (d, *J* = 6.9 Hz, 2H), 7.74 (s, 1H), 7.57–7.40 (m, 4H), 7.31 (d, *J* = 7.1 Hz, 1H), 7.21 (t, *J* = 7.5 Hz, 1H), 6.88 (s, 1H), 3.31–3.24 (m, 2H), 1.75–1.61 (m, 2H), 1.56–1.38 (m, 2H), 0.96 (t, *J* = 7.3 Hz, 3H).

##### 7-((4-Methoxyphenyl)amino)-9-phenylphenanthridin-6(*5H*)-one (**APH2**)

^1^H NMR (250 MHz, CDCl_3_) δ 10.93 (s, 1H), 9.20 (s, 1H), 8.27 (d, *J* = 7.8 Hz, 1H), 7.73 (d, *J* = 1.3 Hz, 1H), 7.63 (dd, *J* = 8.1, 1.5 Hz, 2H), 7.52–7.40 (m, 4H), 7.33–7.27 (m, 3H), 7.26 (d, *J* = 1.4 Hz, 1H), 7.18 (d, *J* = 7.9 Hz, 1H), 6.99 (d, *J* = 8.9 Hz, 2H), 3.88 (s, 3H).

##### 7-((4-Dimethylaminophenyl)amino)-9-phenylphenanthridin-6(*5H*)-one (**APH3**)

^1^H NMR (250 MHz, DMSO-*d*_6_) δ 8.46 (d, *J* = 8.1 Hz, 1H), 7.84 (s, 1H), 7.68 (d, *J* = 6.9 Hz, 2H), 7.59–7.42 (m, 5H), 7.42–7.32 (m, 2H), 7.29–7.17 (m, 3H), 7.11 (s, 1H), 6.80 (d, *J* = 8.9 Hz, 2H), 2.91 (s, 5H).

##### 7-((4-Methoxyphenyl)amino)-9-(thiophen-2-yl)phenanthridin-6(*5H*)-one (**APH4**)

^1^H NMR (250 MHz, DMSO-*d*_6_) δ 11.62 (s, 1H), 11.19 (s, 1H), 8.46 (d, *J* = 7.9 Hz, 1H), 7.94 (s, *J* = 1.0 Hz, 1H), 7.74 (dd, *J* = 3.7, 1.0 Hz, 1H), 7.62 (dd, *J* = 5.0, 1.0 Hz, 1H), 7.50 (t, *J* = 8.1 Hz, 1H), 7.38–7.23 (m, 4H), 7.21–7.14 (m, 2H), 7.02 (d, *J* = 8.9 Hz, 2H), 3.79 (s, 3H).

##### 7-(Phenylamino)-9-(thiophen-2-yl)phenanthridin-6(*5H*)-one (**APH5**)

^1^H NMR (250 MHz, DMSO-*d*_6_) δ 11.35 (d, *J* = 9.0 Hz, 2H), 8.64 (d, *J* = 8.1 Hz, 1H), 8.12 (d, *J* = 1.6 Hz, 1H), 7.84 (dd, *J* = 3.7, 1.2 Hz, 1H), 7.77 (d, *J* = 8.3 Hz, 1H), 7.72–7.63 (m, 2H), 7.52–7.45 (m, 2H), 7.45–7.37 (m, 3H), 7.29–7.08 (m, 2H).

### Metal-Chelating Study

The chelating studies were performed with a diode array HP8543 UV–vis spectrophotometer (Agilent Technologies), by using a 1.0 cm quartz cuvette and the HP Chemstation software. The absorption spectra were collected at room temperature. Absorbance measurements of the tested compounds alone or in the presence of FeCl_2_, CuCl_2_, HgCl_2_, ZnCl_2_, CdCl_2_, or AlCl_3_ were carried out in 100 mM phosphate buffer solution at pH 7.30 and registered within the 200–600 nm range. In all cases, blank solutions were properly analyzed.

Stock solutions of Fe^2+^, Cu^2+^, Hg^2+^, Zn^2+^, Cd^2+^, and Al^3+^ (2.5 mM, final concentration) were prepared in Milli-Q water. The metal solutions were stirred until complete dissolution by using an ultrasound probe (Vibracell Sonics), equipped with a 2 mm diameter titanium microtip, at 60% power width during 2 min. Evaluated compounds were dissolved in an aqueous mixture of DMSO 100 mM phosphate buffer pH = 7.30 at ratios 1:2 v/v (0.50 mM, final concentration) and 1:14 v/v (0.10 mM, final concentration) by employing an ultrasonic bath for 10 min. Working solutions of compounds alone and compound-metal complexes were daily prepared by dilution of stock solutions in buffer as required.

Chelation was detected as a consequence of the changes observed in the absorption spectra after 30 min of incubation. With the aim of estimating the stoichiometry of the compound-metal complexes, a fixed amount of **APH1**, **AHP2**, **APH3**, **APH4**, or **APH5** compounds (50 μM) was mixed with increasing amounts of metal ion (6.25–50 μM). Similarly, a fixed amount of metal (50 μM) was mixed with increasing amounts of **APH1**, **AHP2**, **APH3**, **APH4**, or **APH5** compounds to achieve compound concentrations from 50 to 200 μM. UV–vis spectra were carefully examined in order to evaluate absorption differences and to estimate the ratio metal/compound in the formed complexes. Derivative spectroscopy was also applied to resolve stoichiometry studies.

### HOMO and LUMO Analysis

The ability of a molecule to form complexes can be examined by the molecular orbital frontier approach. This approach is based on the premise that electrons are transferred from the HOMO orbital to the LUMO orbital in the formation of new bonds between the atoms. Thus, HOMO orbital indicates the ability to donate electrons, the LUMO orbitals show the ability to accept electrons of a molecule, and the greater the energy gap is between these HOMO/LUMO orbitals, the higher the molecule polarizability is, which is usually translated into a higher chemical reactivity.^50^

Molecular modeling was performed with ChemBio3D software (version 14.0 ultra), and HOMO and LUMO levels were researched using molecular mechanics (MM2) and MMFF94 force field analysis. For the optimized structure, HOMO–LUMO energies were analyzed.

### Physicochemical and Prediction of ADMET Properties Analysis

In silico assessment of relevant drug-likeness properties and a series of ADMET properties was carried out using ADMETsar version 2.0 (http://lmmd.ecust.edu.cn/admetsar2) and SwissADME; S (http://www.swissadme.ch/) software.^51,52^ We calculated the most significant physicochemical properties required for absorption into the central nervous system such as the molecular weight and octanol–water partition coefficient (log *P*), as other parameters in association with Lipinski’s rule of five and the suitability of a drug molecule for oral administration such as H-bond acceptors and H-bond donors.^53^

### Culture of SN56 Cells

The protection induced by **APH** compounds against metals toxic effects was evaluated using a cholinergic murine neuroblastoma cell line (SN56 cells) derived from the basal forebrain septum. Basal forebrain contains the majority of CNS cholinergic neurons,^54^ which innervate the hippocampus and frontal cortex, regulating learning and memory process.^55,56^ Their selective degeneration, as observed in AD and other neurodegenerative diseases, induced cognitive dysfunction.^57,58^ We chose this cells line since the essential and heavy metals brain accumulation was related with memory and learning alterations,^1−3^ with some of them reported to induce a more pronounced damage in basal forebrain cholinergic neurons,^8,59^ so it is a sensitive model to study their toxic effects. Dulbecco’s modified Eagle’s medium (DMEM) supplemented with 10% fetal bovine serum, penicillin/streptomycin, 2 mM l-glutamine (Sigma, Madrid, Spain), and 1 mM sodium pyruvate was used to maintain cells at 37 °C and 5% CO_2_. This medium was changed every 48 h.^60^ Cells were differentiated via culture for 3 days with 1 mM dibutyryl-cAMP and 1 μM retinoic acid.^61,62^ Following cells differentiation, there were no differences in cells regarding the periods of treatment. All cells used in these studies showed to be mycoplasma-free using the Look Out Mycoplasma PCR Detection Kit (Sigma, Madrid, Spain).

Cells were seeded in 6-well plates at a density of 10^6^ cells/well. To determine **APH** compounds neuroprotective effects against FeCl_2_ (200 μM), CuCl_2_ (20 μM), ZnCl_2_ (250 μM), CdCl_2_ (100 μM), HgCl_2_ (30 μM), and AlCl_3_ (300 μM) cytotoxic effects, cells were treated with the **APH1**, **AHP2**, **APH3**, **APH4**, or **APH5** compounds in concentrations between 10 and 100 μM and with trolox at 100 μM concentration. Besides, to determine the antioxidant mechanisms of **APH1**, **AHP2**, **APH3**, **APH4**, and **APH5** compounds, cells were treated for 24 h with them in concentrations between 10 and 100 μM. At least three replicate wells/treatment were used. A vehicle group was employed in parallel for each experiment as a control.

**APH1**–**APH5** compounds were selected because they were the **APH** compounds with less cytotoxic effects and because they presented the most potent antioxidant and neuroprotective effect against ROS stimulus.^17^ The range of 10–100 μM concentrations was chosen because it is a range of concentrations without cytotoxicity in which it is more probable that their neuroprotective effects are developed. The concentrations of metals used were selected because they were observed to induce cell death (data not shown). The metals used were selected because they were reported to induce neurodegeneration and were mainly associated with neurodegenerative disese.^1−3^

### Cytotoxic and Neuroprotective Effect on SN56 Cells

The 3-(4,5-dimethylthiazol-2-yl)-2,5-diphenyltetrazolium bromide (MTT) assay, which is based on the cleavage of the yellow tetrazolium salt MTT to purple formazan crystals by mitochondrial dehydrogenase, was used to evaluate SN56 cells viability. The cytotoxic effects of metals on SN56 cells were determined after incubation for 24 h with FeCl_2_ (200 μM), CuCl_2_ (20 μM), ZnCl_2_ (250 μM), CdCl_2_ (100 μM), HgCl_2_ (30 μM), and AlCl_3_ (300 μM). The metal concentrations used were previously tested and observed to induce cell death (data not shown). The neuroprotective effects of compounds **APH1**, **AHP2**, **APH3**, **APH4**, and **APH5** against these metals were evaluated after incubation for 24 h with FeCl_2_ (200 μM), CuCl_2_ (20 μM), ZnCl_2_ (250 μM), CdCl_2_ (100 μM), HgCl_2_ (30 μM), and AlCl_3_ (300 μM) of SN56 cells pretreated with compounds **4** at various concentrations (10–100 μM) for 2 h.

At the end of the assays, cells were incubated with 100 μL of yellow MTT solution (final concentration 0.5 mg/mL) for 4 h. After 4 h at 37 °C, the medium was removed, and 150 μL DMSO was used to dissolve the formazan reaction product. The formation of solubilized formazan product was measured spectrophotometrically at 570 nm (Fluoroskan Ascent FL Microplate Fluorometer and Luminometer, Thermo Fisher Scientific, Madrid, Spain). Control cells were taken as 100% viability.

### Real-Time PCR Analysis

The Trizol reagent method (Invitrogen, Madrid, Spain) was used to extract total RNA. A Nanodrop 2000 (Thermo Fisher Scientific, Madrid, Spain) spectrophotometer and an Experion Lab Chip (Bio-Rad, Madrid, Spain) gel were used to determine the final RNA concentration and assess the quality of total RNA samples, respectively. We synthesized the first strand cDNA using a PCR array first strand-synthesis kit (C-02; Super Array Bioscience, Madrid, Spain) with 1000 ng of cRNA, following the manufacturer’s instructions, with an added genomic DNA elimination step and external RNA controls. Following reverse transcription, QPCR was performed using prevalidated primer sets (SuperArray Bioscience) for mRNAs encoding *Keap1* (PPM34919B), *Nrf2* (PPM24614A), *Cat* (PPM04394C), *Gpx* (PPM04345E), and *Actb* (PPM02945B). *Actb* was used as an internal control for normalization. A CFX96 was used to run reactions with real-time SYBR green PCR master mix PA-012 (SuperArray Bioscience). The thermocycler parameters were established as 95 °C for 10 min, followed by 40 cycles of 95 °C for 15 s and 72 °C for 30 s. We used the Ct (cycle threshold) method to calculate relative changes in gene expression. The expression data are presented as actual change multiples.^63^

### Protein Expression Analysis

Treated cells were washed with cold PBS, scraped, and harvested. Then, harvested cells were lysed using radioimmunoprecipitation assay (RIPA) reagent (Thermo Fisher, Madrid, Spain) with a protease repressors mixture (Thermo Fisher, Madrid, Spain), following the manufacturer’s protocol. Finally, cell lysates were centrifuged at 10,000*g* during 10 min at 4 °C, and the supernatant was collected. Total protein levels were assayed with a bicinchoninic acid (BCA) assay kit (catalog number 23225; ThermoFisher, Madrid, Spain), according to manufacturer’s guideline.

Keap1, Nrf2, Gpx, and Cat protein expression was determined employing commercial ELISA tests in cell lysate supernatant (catalog numbers MBS7212746, MBS776676, MBS732750, and MBS728474, respectively; MyBiosource, CA, USA) in accordance with producer’s protocols. The protein concentrations of each target gene were normalized with the total protein levels measured by BCA kit, to prevent possible interferences with the real value of the targets’ protein concentrations measured due to the induction of cell death. Protein levels were expressed in ng/mg protein.

### Protective Effects on Cell Lipid Peroxidation

MDA concentration was determined as an indicator of lipid peroxidation products induced by free radicals. Intracellular MDA production was quantified after 24 h exposure to FeCl_2_ (200 μM) or CdCl_2_ (100 μM) with or without **APH1**, **AHP2**, **APH3**, **APH4**, or **APH5** compounds (10–100 μM), using a Lipid Peroxidation MDA Assay Kit (Abcam, Cambridge, UK), following the manufacturer’s protocol. Briefly, following treatment, we collected 1 × 10^6^ cells and homogenized them on ice in MDA lysis buffer (300 μL) with 3 μL BHT (100×). Then, the mix was centrifuged for 10 min at 13,000*g* to remove insoluble material. Sample (200 μL) or standard (200 μL of MDA) was mixed with 600 μL of thiobarbituric acid solution, incubated at 95 °C for 50 min, and cooled to room temperature in an ice bath for 10 min. We loaded all samples and standards (200 μL) (duplicate) into a clear 96-well plate, and a microplate reader (Fluoroskan Ascent FL Microplate Fluorometer and Luminometer, Thermo Fisher Scientific, Madrid, Spain) was used to record the absorbance at 532 nm. Concentration of MDA, determined as nmol/mg protein, is presented as percent untreated control.

### Statistical Analysis

At least three replicates for each experimental condition were performed, and the presented results were representative of these replicates. Data are presented as means ± standard error of the mean. Comparisons between experimental and control groups were performed by two-ways ANOVA analyses (concentration vs treatment) or one-way ANOVA analyses (analysis of different 7-aminophenanthridin compounds concentrations) followed by the Tukey posthoc test. Statistical difference was accepted when *p* ≤ 0.05. Statistical analysis of data was carried out using GraphPad Prism 5.01 software.

## Supporting Information Available

Figures showing optimized molecular structure of **APH1**–**APH3** and **APH5**, their iron complex and their corresponding HOMOs and LUMOs; cell viability effects after **APH1**–**APH5** treatment; and **APH1**–**APH5** UV–vis spectra and first-order derivative absorption spectra, alone and in the presence of metals. Tables with results of HOMO/LUMO gap energy analysis and ADMET prediction of APH compounds (PDF)

## Abbreviations

AD: Alzheimer’s disease
ADMET: absorption, distribution, metabolism, excretion, and toxicological properties
ALS: amyotrophic lateral sclerosis
ANOVA: analysis of variance
APH: 7-amino-phenanthridin-6-one derivatives
BBB: blood–brain barrier
BHT: butylated hydroxytoluene
CNS: central nervous system
DDQ: 2,3-dichloro-5,6-dicyano-1,4-benzoquinone
DMEM: Dulbecco’s modified Eagle’s medium
DMSO: dimethyl sulfoxide
HD: Huntington’s disease
HOMO: Highest occupied molecular orbital
KEAP1: Kelch-like ECH-associated protein 1
LUMO: Lowest unoccupied molecular orbital
MDA: malondialdehyde
MMP: matrix metalloproteinase
MMFF94: Merck molecular force field 94
MM2: molecular mechanics force field
MTT: 3-(4,5-dimethylthiazol-2-yl)-2,5-diphenyltetrazolium
NRF2: nuclear factor erythroid 2-related factor 2
PARP-1: poly(ADP-ribose) polymerase-1
PD: Parkinson’s disease
p-tau: tau-phosphorylated
ROS: reactive oxygen species
UV–vis: ultraviolet–visible spectroscopy
