# Supplementary material for: Neuroprotective Action of Multitarget 7-Aminophenanthridin-6(5H)-one Derivatives against Metal-Induced Cell Death and Oxidative Stress in SN56 Cells
Source: ACS Chem Neurosci. 2021 Aug 30;12(18):3358–72. doi: 10.1021/acschemneuro.1c00333 (PMC8478279; doi:10.1021/acschemneuro.1c00333)
Supplement: Supplementary file 1 — cn1c00333_si_001.pdf [file cn1c00333_si_001.pdf]

Supporting Information

Neuroprotective action of multitarget 7-aminophenanthridin-6(*5H*)-one derivatives against metal-induced cell death and oxidative stress in SN56 cells.

Paula Moyano,[1,‡] David Vicente-Zurdo,[2,‡] Cristina Blázquez-Barbadillo,[3] J. Carlos Menéndez,[3] Juan F. González,[3]* Noelia Rosales-Conrado,[2]* and Javier del Pino[1,]*

[1]Departamento de Farmacología y Toxicología, Facultad de Veterinaria, Universidad Complutense, 28040 Madrid, Spain.

[2]Departamento de Química Analítica, Facultad de Ciencias Químicas, Universidad Complutense, 28040 Madrid, Spain.

[3]Unidad de Química Orgánica y Farmacéutica, Departamento de Química en Ciencias Farmacéuticas, Facultad de Farmacia, Universidad Complutense, 28040 Madrid, Spain.

[‡]Authors with equal contribution

Running Title: Neuroprotective action of 7-aminophenanthridin-6(*5H*)-one derivatives against metals.

***Corresponding authors**:

J. Del Pino jdelpino@pdi.ucm.es
N. Rosales-Conrado nrosales@ucm.es
J.F. González jfgonzal@ucm.es

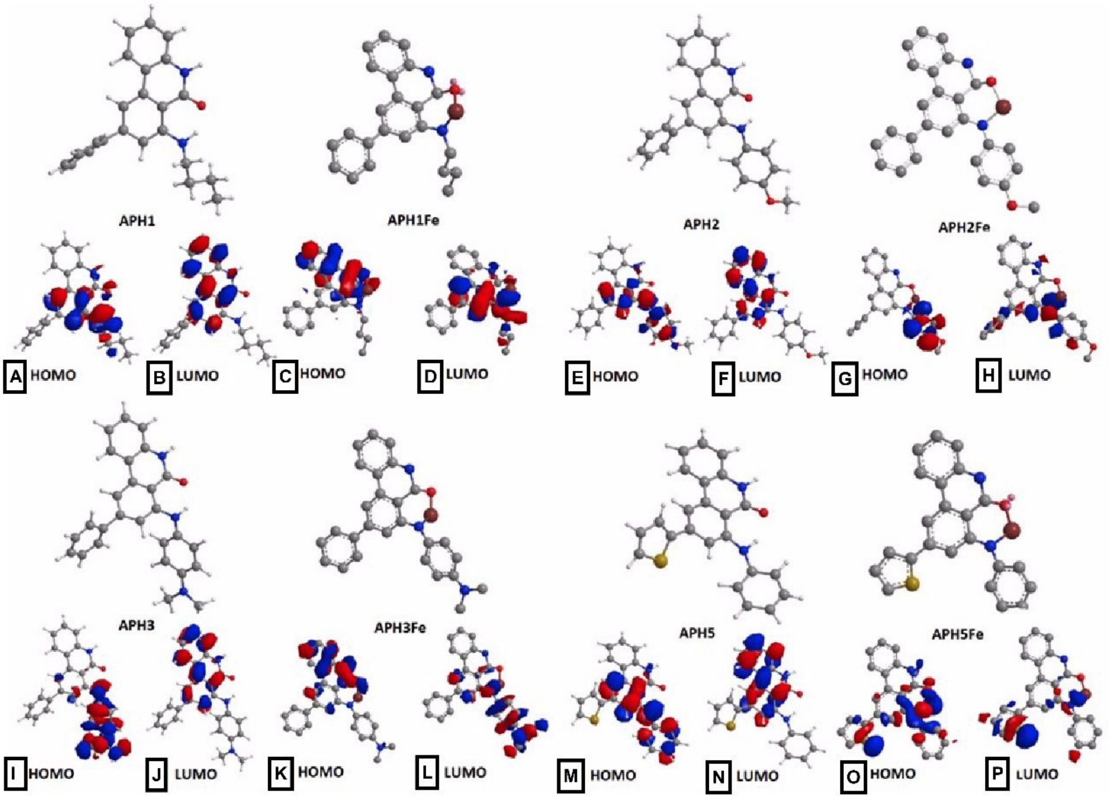

Figure S1. Optimized molecular structure of the **APH1**, **APH2**, **APH3** and **APH5** compounds, their iron complex (upper) and their corresponding HOMOs (A, C, E, G, I, K, M and O) and LUMOs (B, D, F, H, J, L, N and P). Color scheme: carbons is gray, hydrogen is white, nitrogen is blue, oxygen is red, sulphur is yellow, and iron is brown. HOMOs and LUMOs are shown in red and blue.

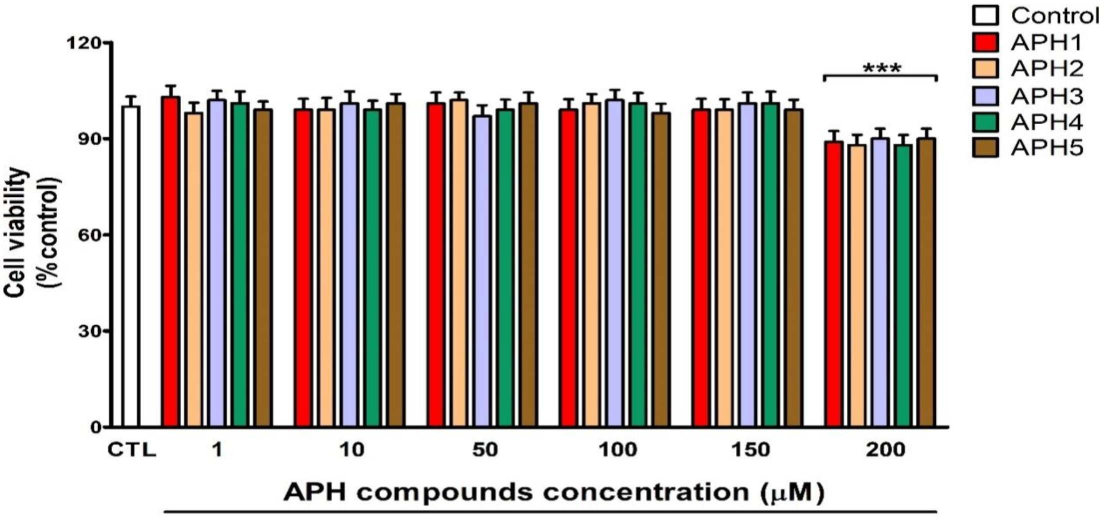

Figure S2. Cell viability effects after APH compounds (10-100 μM) treatment. Data represents the mean ± SEM of three independent experiments in triplicate. $^{***}p \leq 0.001$ compared to control.

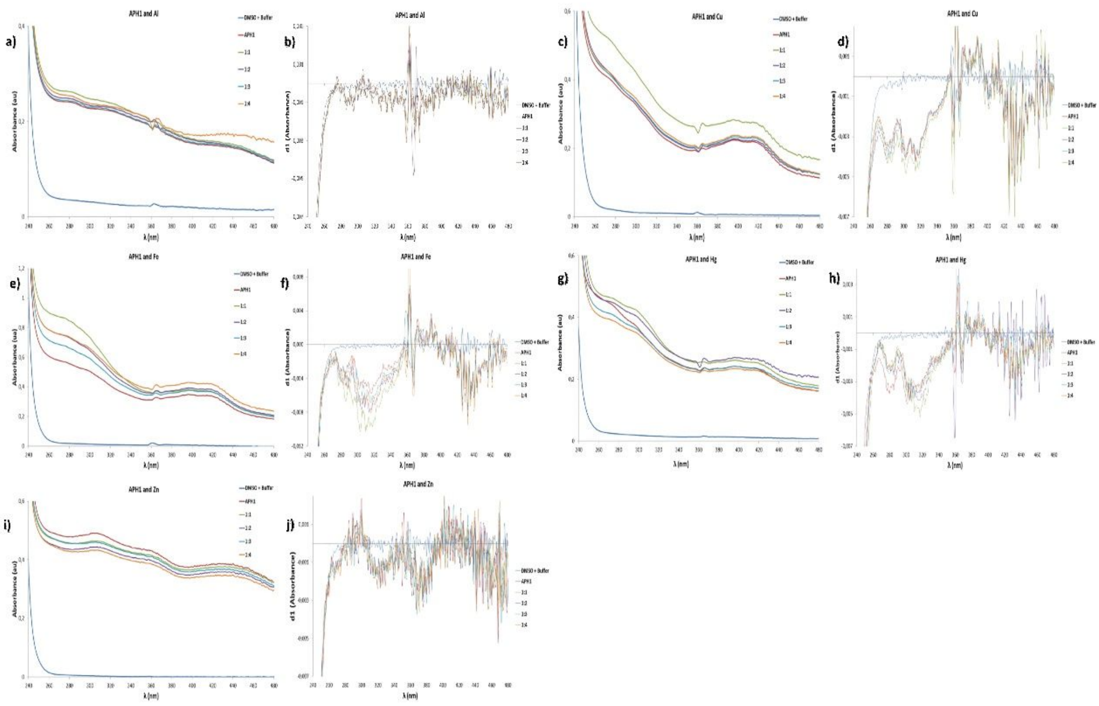

Figure S3. UV-Vis spectra (a, c, e, g and i) and first-order derivative absorption spectra (b, d, f, h and j) of **APH1** alone and in the presence of metals in buffer (100 mM phosphate, pH= 7.30) at room temperature. [**APH1**] = 50 μM and [Metal] = 50, 25, 12.5 and 6.25 μM corresponding to stoichiometries 1:1, 1:2, 1:4 and 1:8, respectively.

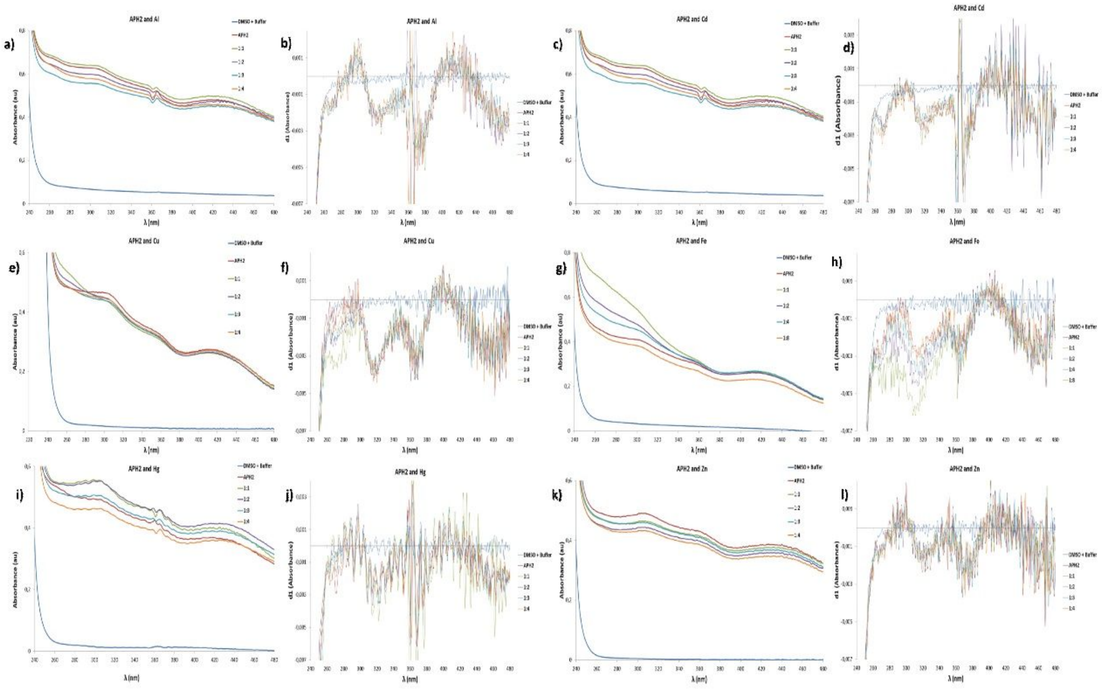

Figure S4. UV-Vis spectra (a, c, e, g, i and k) and first-order derivative absorption spectra (b, d, f, h, j and l) of **APH2** alone and in the presence of metals in buffer (100 mM phosphate, pH= 7.30) at room temperature. [**APH2**] = 50 μM and [Metal] = 50, 25, 12.5 and 6.25 μM corresponding to stoichiometries 1:1, 1:2, 1:4 and 1:8, respectively.

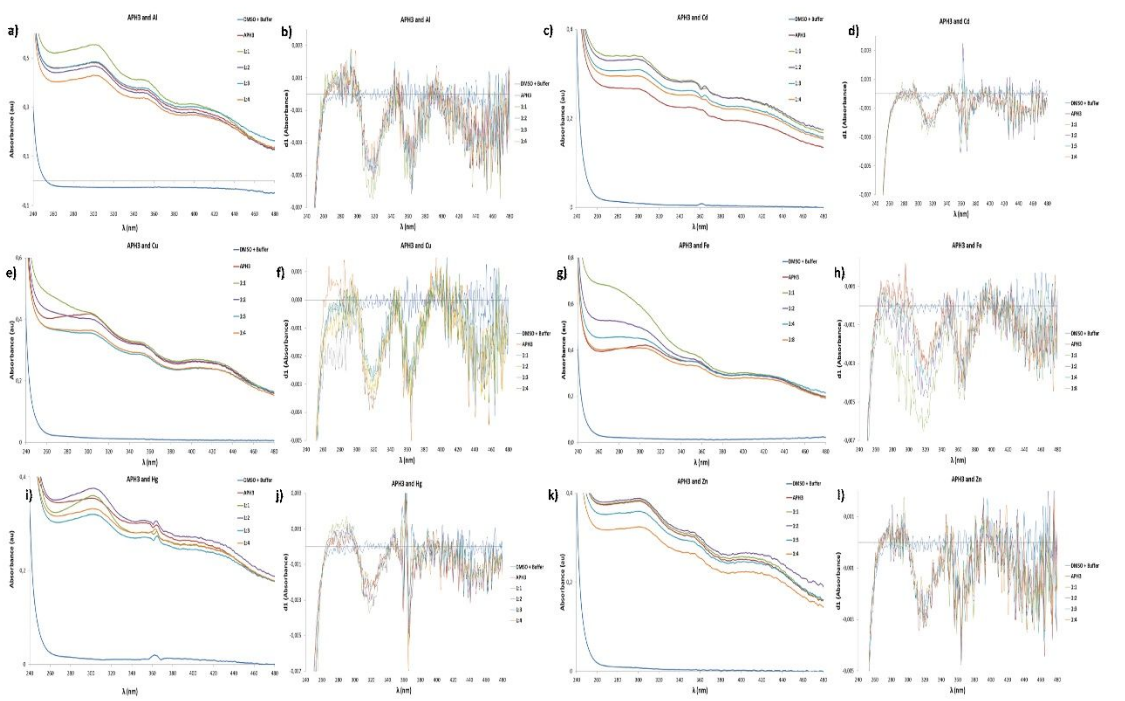

Figure S5. Figure S4. UV-Vis spectra (a, c, e, g, i and k) and first-order derivative absorption spectra (b, d, f, h, j and l) of **APH3** alone and in the presence of metals in buffer (100 mM phosphate, pH= 7.30) at room temperature. [**APH3**] = 50 µM and [Metal] = 50, 25, 12.5 and 6.25 µM corresponding to stoichiometries 1:1, 1:2, 1:4 and 1:8, respectively.

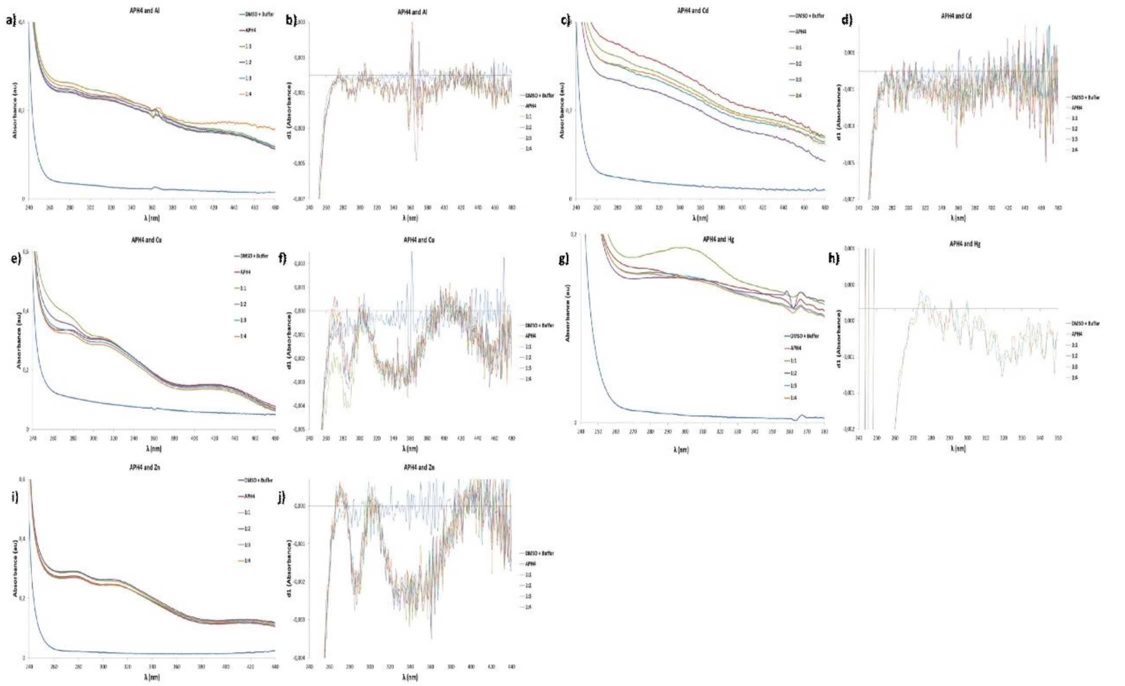

Figure S6. UV-Vis spectra (a, c, e, g and i) and first-order derivative absorption spectra (b, d, f, h and j) of **APH4** alone and in the presence of metals in buffer (100 mM phosphate, pH= 7.30) at room temperature. [**APH4**] = 50 µM and [Metal] = 50, 25, 12.5 and 6.25 µM corresponding to stoichiometries 1:1, 1:2, 1:4 and 1:8, respectively.

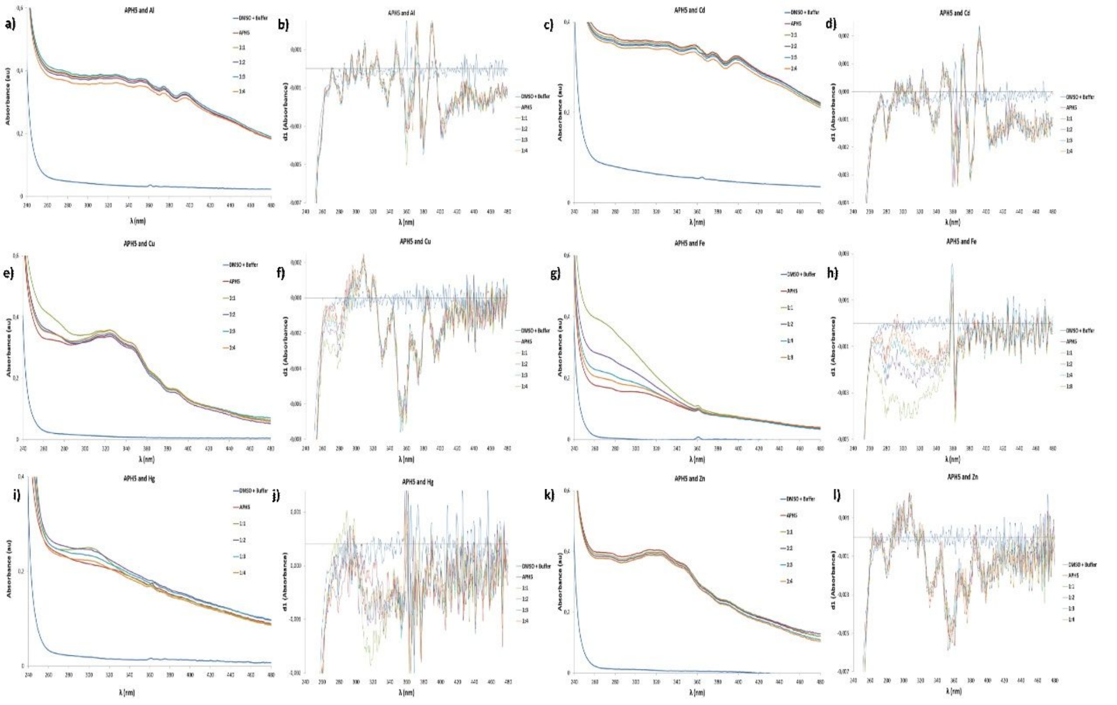

Figure S7. UV-Vis spectra (a, c, e, g, i and k) and first-order derivative absorption spectra (b, d, f, h, j and l) of **APH5** alone and in the presence of metals in buffer (100 mM phosphate, pH= 7.30) at room temperature. [**APH5**] = 50 µM and [Metal] = 50, 25, 12.5 and 6.25 µM corresponding to stoichiometries 1:1, 1:2, 1:4 and 1:8, respectively.

Table S1. HOMO/LUMO gap energy analysis.

Compounds	LUMO (eV)	HOMO (eV)	Gap (eV)
APH1	-3.386	-10.435	7.049
APH2	-3.361	-11.160	7.799
APH3	-3.543	-8.037	4.494
APH4	-3.456	-9.438	5.982
APH5	-3.652	-10.295	6.643
APH1Fe	-10.205	-11.122	0.917
APH2Fe	-9.051	-9.763	0.712
APH3Fe	-7.018	-10.940	3.922
APH4Fe	-7.450	-7.595	0.145
APH5Fe	-7.419	-7.718	0.299

Table S2. ADMET prediction of APH compounds.

	APH1	APH2	APH3	APH4	APH5
Caco-2 permeability Value/Probability	Yes, (0.66)[a]	Yes, (0.71)[a]	Yes, (0.71)[a]	Yes, (0.66)[a]	Yes, (0.67)[a]
GI absorption Value/Probability	Yes, (0.98)[a]; High[b]	Yes, (0.98)[a]; High[b]	Yes, (0.98)[a]; High[b]	Yes, (0.97)[a]; High[b]	Yes, (0.96)[a]; High[b]
BBB permeant Value/Probability	Yes, (0.99)[a]; Yes[b]	Yes, (0.98)[a]; No[b]	Yes, (0.99)[a]; No[b]	Yes, (0.98)[a]; No[b]	Yes, (0.99)[a]; No[b]
CYP1A2 inhibitor Value/Probability	Yes, (0.82)[a]; Yes[b]	Yes, (0.79)[a]; Yes[b]	Yes, (0.79)[a]; Yes[b]	Yes, (0.82)[a]; Yes[b]	Yes, (0.82)[a]; Yes[b]
CYP2C19 inhibitor Value/Probability	No, (0.53)[a]; Yes[b]	Yes, (0.84)[a]; Yes[b]	No, (0.81)[a]; Yes[b]	Yes, (0.93)[a]; Yes[b]	Yes, (0.53)[a]; Yes[b]
CYP2C9 inhibitor Value/Probability	No, (0.59)[a]; No[b]	Yes, (0.84)[a]; No[b]	No, (0.80)[a]; No[b]	Yes, (0.93)[a]; Yes[b]	Yes, (0.53)[a]; No[b]
CYP2D6 substrate Value/Probability	No, (0.67)[a]; Yes[b]	No, (0.82)[a]; No[b]	No, (0.84)[a]; No[b]	No, (0.83)[a]; Yes[b]	No, (0.80)[a]; No[b]
P glycoprotein substrate Value/Probability	No, (0.70)[a]; Yes[b]	No, (0.83)[a]; Yes[b]	No, (0.76)[a]; Yes[b]	No, (0.58)[a]; Yes[b]	No, (0.88)[a]; Yes[b]

[a]Prediction with ADMETsar version 2 (Probability). [b]Prediction with Swiss ADME.
